# Inflammatory markers in depression: A meta-analysis of mean differences and variability in 5,166 patients and 5,083 controls

**DOI:** 10.1016/j.bbi.2020.02.010

**Published:** 2020-07

**Authors:** Emanuele F. Osimo, Toby Pillinger, Irene Mateos Rodriguez, Golam M. Khandaker, Carmine M. Pariante, Oliver D. Howes

**Affiliations:** a MRC London Institute of Medical Sciences, Faculty of Medicine, Imperial College London, Hammersmith Hospital Campus, London, UK; b Department of Psychiatry, University of Cambridge, Cambridge, UK; c Cambridgeshire and Peterborough NHS Foundation Trust, Cambridge, UK; d Institute of Psychiatry, Psychology and Neuroscience, King’s College London, London, UK; e School of Clinical Medicine, University of Cambridge, Cambridge, UK; f National Institute for Health Research, Mental Health Biomedical Research Centre, South London and Maudsley NHS Foundation Trust and King's College London, London, UK; g The Maurice Wohl Clinical Neuroscience Institute, Cutcombe Road, London SE5 9RT, UK

**Keywords:** Depression, Inflammation, Meta-analysis, Heterogeneity, Cytokine, CRP

## Abstract

**Importance:**

The magnitude and variability of cytokine alterations in depression are not clear.

**Objective:**

To perform an up to date meta-analysis of mean differences of immune markers in depression, and to quantify and test for evidence of heterogeneity in immune markers in depression by conducting a meta-analysis of variability to ascertain whether only a sub-group of patients with depression show evidence of inflammation.

**Data Sources:**

Studies that reported immune marker levels in peripheral blood in patients with depression and matched healthy controls in the MEDLINE database from inception to August 29th 2018 were examined.

**Study Selection:**

Case-control studies that reported immune marker levels in peripheral blood in patients with depression and healthy controls were selected.

**Data Extraction and Synthesis:**

Means and variances (SDs) were extracted for each measure to calculate effect sizes, which were combined using multivariate meta-analysis.

**Main Outcomes and Measures:**

Hedges *g* was used to quantify mean differences. Relative variability of immune marker measurements in patients compared with control groups as indexed by the coefficient of variation ratio (CVR).

**Results:**

A total of 107 studies that reported measurements from 5,166 patients with depression and 5,083 controls were included in the analyses. Levels of CRP (*g* = 0.71; 95%CI: 0.50–0.92; *p* < 0.0001); IL-3 (*g* = 0.60; 95%CI: 0.31–0.89; *p* < 0.0001); IL-6 (*g* = 0.61; 95%CI: 0.39–0.82; *p* < 0.0001); IL-12 (*g* = 1.18; 95%CI: 0.74–1.62; *p* < 0.0001); IL-18 (*g* = 1.97; 95%CI: 1.00–2.95; *p* < 0.0001); sIL-2R (*g* = 0.71; 95%CI: 0.44–0.98; *p* < 0.0001); and TNFα (*g* = 0.54; 95%CI: 0.32–0.76; *p* < 0.0001) were significantly higher in patients with depression. These findings were robust to a range of potential confounds and moderators. Mean-scaled variability, measured as CVR, was significantly lower in patients with depression for CRP (CVR = 0.85; 95%CI: 0.75–0.98; *p* = 0.02); IL-12 (CVR = 0.61; 95%CI: 0.46–0.80; *p <* 0.01); and sIL-2R (CVR = 0.85; 95%CI: 0.73–0.99; *p* = 0.04), while it was unchanged for IL-3, IL-6, IL-18, and TNF α.

**Conclusions and Relevance:**

Depression is confirmed as a pro-inflammatory state. Some of the inflammatory markers elevated in depression, including CRP and IL-12, show reduced variability in patients with depression, therefore supporting greater homogeneity in terms of an inflammatory phenotype in depression. Some inflammatory marker elevations in depression do not appear due to an inflamed sub-group, but rather to a right shift of the immune marker distribution.

## Highlights

• Patients with depression show reduced variability in pro-inflammatory immune measures.
• Patients with depression show increases in pro-inflammatory immune markers mean levels, and reductions in anti-inflammatory IL-4.

## 1 Introduction

Depression is a common mental illness and is one of the leading causes of disability worldwide, affecting around 10–20% of the general population in their lifetime (Lim et al., 2018). A better understanding of the pathophysiology of depression is required to identify novel therapeutic targets to improve treatment (Maes et al., 2012). Converging lines of evidence suggest immune dysregulation plays a role in the pathogenesis of depression: early-life infection and autoimmune diseases are associated with a higher risk of depression in adulthood (Benros et al., 2013). Direct evidence of inflammation in depression comes from meta-analyses of cross-sectional studies of inflammatory markers in depression, which have shown increased concentrations of circulating C-reactive protein (CRP), interleukin 6 (IL-6), interleukin-12 (IL-12), tumor necrosis factor-α (TNFα), and reductions in interleukin-4 (IL-4) in acute depression (Howren et al., 2009, Dowlati et al., 2010, Haapakoski et al., 2015, Goldsmith et al., 2016, Köhler et al., 2017). Further evidence for a role of inflammation in psychiatric disorders comes from treatment studies: meta-analyses of clinical trials indicate that anti-inflammatory drugs may have antidepressant effects (Köhler et al., 2014, Kappelmann et al., 2016).

However, it has been proposed that inflammation is a factor only for some patients with depression (Khandaker et al., 2017). Supporting this, some studies have found that higher cytokine levels are only seen in a proportion of patients with depression (Benedetti et al., 2002, Lanquillon et al., 2000, Carvalho et al., 2013); in particular, treatment resistant patients show greater elevations in CRP than treatment responsive patients (Chamberlain et al., 2019). Moreover, it has been shown that inflammatory levels tend to normalise in most patients following recovery, while raised inflammatory markers do not normalise in treatment resistant patients (Maes et al., 1997, O’Brien et al., 2007). Further support to the importance of immune factors in treatment response in depression is that a number of trials have shown a lack of efficacy of anti-inflammatories in depression, and have suggested this variability may be due to heterogeneity in the inflammatory alterations amongst patients with depression (Kappelmann et al., 2018, Raison et al., 2013). Finally, there are ongoing clinical trials in people with depression and an inflamed phenotype at baseline, testing if specific inflammatory cytokines such as IL-6 contribute to the pathogenesis of this type of “inflamed depression”, and if their clinical phenotype differs from people with “non-inflamed” depression (Khandaker et al., 2018). Therefore, individual variability in the peripheral immune marker phenotype might be both contributing to shaping the clinical phenotype, and also to affect outcomes, such as treatment response. Thus, determining if there is evidence for heterogeneity in inflammatory markers in depression is important to determine if clinical trials need to target specific patients, or if inflammation is a general component of the pathophysiology of depression.

Heterogeneity can be systematically compared relative to controls in a meta-analysis of variability (Brugger and Howes, 2017, Pillinger et al., 2018:). To our knowledge no previous meta-analysis has investigated variability in inflammatory cytokines in depression.

Another important issue is that smoking and high BMI, which are common in major depression (Dierker et al., 2002, Stunkard et al., 2003, Anda et al., 1990), can significantly affect peripheral inflammatory marker levels (Nieman et al., 1999, Brooks et al., 2010, Trayhurn and Wood, 2004, Yanbaeva et al., 2007). However, few of the available meta-analyses of immune markers in depression have systematically considered the effect of clinical confounds such as smoking or high BMI on immune alterations (Supplementary Table 1). Thus, it remains unclear to what degree the association between depression and peripheral inflammatory markers is secondary to smoking or high BMI.

We therefore set out to:

Main objective: quantify and test for evidence of heterogeneity in immune markers in depression by conducting a meta-analysis of variability (Brugger and Howes, 2017, Pillinger et al., 2018:);

Secondary objective: perform an up-to-date meta-analysis of mean levels of cytokines in depression, taking into account smoking, high BMI and other potential clinical and demographic confounds.

## 2 Methods

### 2.1 Search strategy and study selection

The Pubmed, EMBASE, and PsycINFO databases were independently searched for studies investigating CRP, cytokines, TNFα, transforming growth factor (TGF) and interferon levels in patients with depression and healthy controls. The search was complemented by hand-searching of meta-analyses and review articles.

### 2.2 Data extraction and processing

We extracted means and variance measures (SDs) of immune parameters for the patient and control groups. In addition, we recorded details of the following potential moderating factors: age, gender, ethnicity, BMI and smoking status.

### 2.3 Statistical analysis

Original study data was reported as raw or log-transformed in different original studies; data was converted to raw as needed using the formula in (Higgins et al., 2008). As many studies reported on several parameters, multivariate meta-analysis was used, enabling simultaneous estimation of summary effect sizes across all immune parameters, and reducing risk of false positives due to multiple comparisons (Bender et al., 2008). For all meta-analyses, an omnibus test evaluated significance of model coefficients across immune parameters. Where the omnibus test was significant, we moved on to multivariate meta-analysis in order to test the effect separately for each parameter.

A meta-analysis of between group differences in immune parameters was performed, indexed using Hedges *g*. A random effects model was used owing to expectation of inconsistency across studies.

To measure variability, the natural log of the ratio of estimates of the population standard deviations for each group was calculated to give the log variability ratio (VR), as previously described (Brugger and Howes, 2017, Pillinger et al., 2018:). In biological systems, variance often scales with mean (Eisler et al., 2008). Thus, between group differences in relative variability may, at least partially, be a function of between-group differences in mean. Therefore, a meta-analysis of relative variability of patient compared with control immune parameters scaled to group means was performed: the log coefficient of variation ratio (CVR) (the natural logarithm of the ratio of estimates of population coefficients of variation).

### 2.4 Moderator and sensitivity analyses

We investigated the potential effects of clinical variables (including medication status, duration of illness and whether patients were experiencing a current depressive episode at the time of blood sampling) on the meta-analytic results where this information was available. Due to data availability, the *untreated* category includes both antidepressant-naïve and antidepressant-free patients, with no minimum washout period. To determine if findings were influenced by potential confounds, we performed sensitivity analyses to determine if findings remained in studies that matched patients and controls for age, BMI, smoking levels. We also performed sensitivity analyses based on those reporting measures from serum or plasma, on fresh or frozen samples, on ELISA/multiplex-based assays, and excluding poor quality studies.

Publication bias was assessed for mean differences in all parameters by visual inspection of funnel plots of standard errors against immune residuals. Inconsistency between studies was assessed using the I^2^ statistic (Higgins et al., 2003).

Further details of the search, study selection, data processing and statistical analyses are provided in Supplementary Methods.

## 3 Results

### 3.1 Study selection

We retrieved 9,897 citations, and 9,526 were excluded after title/abstract review. Following manuscript review, 269 studies were excluded based on failure to meet inclusion criteria. The final data set included 107 studies (Lanquillon et al., 2000, Maes et al., 1997, O’Brien et al., 2007, Alcocer-Gómez et al., 2014, Alesci et al., 2005, Ali et al., 2018, Bai et al., 2014, Basterzi et al., 2005, Berk et al., 1997, Boettger et al., 2010, Brambilla and Maggioni, 1998, Camardese et al., 2011, Cassano et al., 2017, Chamberlain et al., 2018, Charlton et al., 2018, Chavda et al., 2011, Chen et al., 2017, Crnković et al., 2012, Dannehl et al., 2014, Davami et al., 2016, Dhabhar et al., 2009, Dinan et al., 2009, Diniz et al., 2010, Diniz et al., 2010, Dome et al., 2009, Dunjic-Kostic et al., 2013, Elderkin-Thompson et al., 2012, Eller et al., 2008, Elomaa et al., 2012, Euteneuer et al., 2011, Euteneuer et al., 2012, Fan et al., 2017, Fornaro et al., 2011, Fornaro et al., 2013, Frodl et al., 2012, Frommberger et al., 1997, Gazal et al., 2015, Goyal et al., 2017, Grosse et al., 2016, Häfner et al., 2008, Hernández et al., 2008, Ho et al., 2017, Hocaoglu et al., 2012, Huang and Lin, 2007, Hosseini et al., 2007, Hughes et al., 2012, Hung et al., 2007, Kageyama et al., 2018, Kagaya et al., 2001, Jozuka et al., 2003, Karlović et al., 2012, Kahl et al., 2015, Kéri et al., 2014, Leo et al., 2006, Lehto et al., 2010, Lehto et al., 2010, Lee and Kim, 2006, Lee et al., 2009, Kubera et al., 2000, Kokai et al., 2002, Kling et al., 2007, Kim et al., 2002, Maes et al., 1990, Miller et al., 2005, Mikova et al., 2001, Merendino et al., 2002, Marques-Deak et al., 2007, Manoharan et al., 2016, O'donovan A, Rush G, Hoatam G, Hughes BM, McCrohan A, Kelleher C, , 2013, O'brien SM, Scott LV, Dinan TG. , 2006, Nunes et al., 2011, Narita et al., 2006, Munjiza et al., 2018, Motivala et al., 2005, Mota et al., 2013, Piletz et al., 2009, Pike and Irwin, 2006, Pavón et al., 2006, Owen et al., 2001, Ogłodek, 2018, Tuglu et al., 2003, Thomas et al., 2005, Sutcigil et al., 2008, Sowa-Kućma et al., 2018, Sluzewska et al., 1996, Simon et al., 2008, Seidel et al., 1995, Schlatter et al., 2004, Savitz et al., 2015, Rybka et al., 2013, Rudzki et al., 2017, Rudolf et al., 2014, Rizavi et al., 2016, Rief et al., 2001, Rawdin et al., 2013, Sugimoto et al., 2018, Shen et al., 2010, Schmidt et al., 2014, Zoga et al., 2014, Zincir et al., 2016, Zou et al., 2018, Yoshimura et al., 2010, Yoshimura et al., 2009, Yang et al., 2007, Xia et al., 2018, Wiener et al., 2017, Vaccarino et al., 2008, Wang et al., 2018, Rapaport and Irwin, 1996), covering data on 306 immune measures (Supplementary Fig. 1 and Supplementary Table 2). The number of measures exceeds the number of studies because in some studies subjects had more than one measure, but we adjusted for multiple measures in the analysis (as discussed in the statistical methods). The total sample consisted of 10,249 people (5,166 patients, 5,083 controls), allowing meta-analysis of: IL-1α, IL-1β, IL-2, IL-3, IL-4, IL-5, IL-6, IL-7, IL-8, IL-10, IL-12, IL-13, IL-18, sIL-1RA, sIL-2R, sIL-6R, TNF α, IFNγ, TGF β, and CRP. The average patient age was 42.53 years, SD 10.94; the median percentage of male patients was 35%, inter-quartile range 22%.

### 3.2 Proportion of skewed data

Prior to log-transformation, there was strong evidence of skew in 122 out of 306 (39.9%) raw-scaled immune measures. This proportion reduced to 117 out of 306 (38.2%) with log-transformation. For all immune parameters, there was no significant difference in the proportion of immune measures with skew in patients compared with controls, either in raw-scaled (OR = 0.72, *p* = 0.08) or log-transformed data sets (OR = 1.00, *p* = 1.00).

### 3.3 Mean differences

**Fig. 1:**
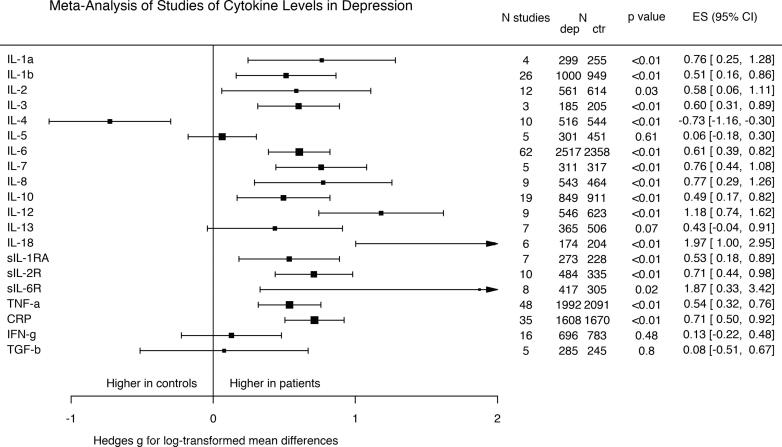
Forest plot showing effect sizes for mean differences in immune parameters in depression compared with healthy controls. *There were significantly higher levels in patients with depression compared with controls for IL-1* α; *IL-1* β*; IL-2; IL-3; IL-6; IL-7; IL-8; IL-10; IL-12; IL-18; IL-1Ra; IL-2R; IL-6R; TNF* α*; and CRP. There was a significant reduction in IL-4 in patients compared with controls. There was no significant difference in TGF β; IFNγ; IL-13; IL-5 in patients compared with controls.*

We found a significant overall effect of group on mean concentration across all immune parameters (omnibus χ^2^ = 217.2, *p* < 0.0001). Fig. 1 shows that significant elevations in the following parameters were observed in depression: IL-1 α; IL-1 β; IL-2; IL-3; IL-6; IL-7; IL-8; IL-10; IL-12; IL-18; IL-1Ra; IL-2R; IL-6R; TNF α; and CRP. A significant reduction in IL-4 was observed in depression. There were no significant differences between groups for: IL-5; IL-13; IFNγ; and TGF β.

#### 3.3.1 Sensitivity analyses of the influence of psychiatric, clinical and lifestyle predictors on mean differences

Supplementary Table 3 shows the results of sensitivity analyses of psychiatric clinical predictors on mean differences in immune parameters in depression compared with healthy controls. Most parameters showed concordance with the main analysis in sensitivity analyses, with the exception of IL-5, which showed a significant elevation in untreated patients, and of IL-13, which showed a significant elevation in the subset of patients experiencing an active depressive episode. Duration of illness was a significant moderator in analyses of IL-5, IL-7, IL-8 and IFNγ. Full details can be found in Supplementary results and in Supplementary Figs 2 and 3.

Supplementary Table 4 shows the results of sensitivity analyses of lifestyle and medical clinical predictors for mean differences in immune parameters in depression compared with healthy controls. Parameters which did not show concordance with the main analysis were IL-2 and IL-8, which were reduced, and TGF β, which was increased in studies matched for BMI. IFNγ showed an increase in patients of studies matched for smoking. Results for age where all concordant with the main analysis. Full details can be found in Supplementary results and in Supplementary Figs. 4–6.

#### 3.3.2 Sensitivity analyses of the influence of skew, publication bias, sample type and study inconsistency

Supplementary Fig. 7 shows results after removing studies with evidence of persistent severe skew despite log transformation. Results not concordant with the main analysis were seen for TGF β, which showed an increase in patients, and for IL-8, which showed a decrease in patients, while IL-1 β’s increase was no longer significant.

A meta-regression taking into-account sample type (both plasma or serum, and fresh or frozen sample) created a data set of 84 studies (4,419 patients, 4,251 controls); 63 studies (75%) utilised measures from serum, while 73 measures (87%) were taken from previously frozen samples. Results were concordant with the main analysis for IL-1 β, IL-2, IL-4, IL-5, IL-6, IL-12, IL-13, sIL-1RA, sIL-2R, TNF α, CRP, TGF β and IFNγ. For IL-7, IL-8, IL-10 and sIL-6R results were no longer significant in this sensitivity analysis. Full details can be found in Supplementary results.

The funnel plot for publication bias demonstrated symmetry (Supplementary Fig. 8), with one outlier (Camardese et al., 2011). Re-analysis with the outlier excluded (Supplementary Fig. 9) showed that results for IL-6R were no longer significant. Higgins’ I^2^ inconsistency values (Supplementary Table 5) demonstrated a medium-large degree of inconsistency for all parameters.

### 3.4 Variability meta-analysis

**Fig. 2:**
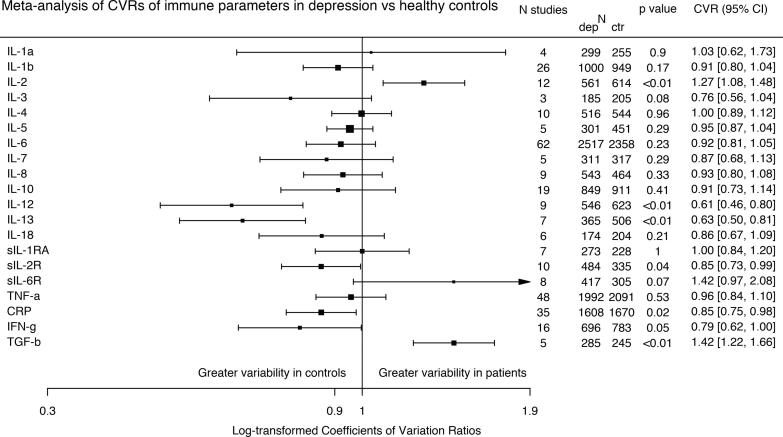
Forest plot showing effect sizes for mean-scaled variability of immune parameters in depression compared with healthy controls. *The coefficient of variation ratio (CVR) was significant decreased for IL-12, IL-13, IL-2R, CRP, and IFN*γ*, indicating lower variability of these immune parameters in patients compared with controls, and significantly increased for IL-2 and TGF* β*, indicating increased variability of these immune parameters in patients compared with controls.*

Given that most cytokines are increased in depression, and variance often scales with mean (Eisler et al., 2008), differences in relative variability may, at least partially, be a function of between-group differences in mean. Therefore, variability ratio results are presented in Supplementary results and Supplementary Fig. 10. Here we present mean scaled coefficient of variation ratios (CVR). We found a significant overall effect of group on log variability ratio across all immune parameters (omnibus χ^2^ = 72.1, p < 0.0001). Fig. 2 shows that there was significantly lower CVR in patients using for IL-12; IL-13; sIL-2R; CRP; and IFNγ. There was no significant difference found in CVR of IL-1 α; IL-1 β; IL-3; IL-4; IL-5; IL-6; IL-7; IL-8; IL-10; IL-18; IL-1RA; IL-6R; and TNF. Analysis of IL-2 and of TGF β showed both to be more variable in patients according to CVR analysis.

#### 3.4.1 Study quality

Newcastle Ottawa Scale quality scores ranged from 3 to 8 (Supplementary Table 6). Of the 107 studies, 19 were rated as ‘poor-quality’. Following the exclusion of these poor-quality studies, meta-analyses could not be carried out for IL-1 α and IL-3, while results of the primary meta-analyses for IL-2, IL-7, IL-8, IL-13, IL-1RA and IL-6R became non-significant, with implications for reliability of these outcomes (Supplementary Fig. 11).

Excluding poor quality studies, results of the CVR meta-analyses for IL-2R and CRP became non-significant, while IL-8 showed greater variability in controls, and IL-18 and IL-1RA showed greater variability in patients (Supplementary Fig. 12).

Table 1 summarizes the main findings of this meta-analysis, while Supplementary Table 7 summarizes all findings.

**Table 1:** Summary of Significant Findings. *The following table summarises the findings of variability and mean differences meta-analyses of inflammatory markers in depression concordant between the main and sensitivity analyses*.

marker	Meta-analysis of mean differences in immune parameters in depression compared with healthy controls	Meta-analysis of variability: CVR
CRP	↑ in patients *g* = 0.71; 95%CI: 0.50–0.92	↓ variability in patients CVR = 0.85; 95%CI: 0.75–0.98
IL-3	↑ in patients *g* = 0.60; 95%CI: 0.31–0.89	↔ CVR = 0.76; 95%CI: 0.56–1.04
IL-6	↑ in patients *g* = 0.61; 95%CI: 0.39–0.82	↔ CVR = 0.92; 95%CI: 0.81–1.05
IL-12	↑ in patients *g* = 1.18; 95%CI: 0.74–1.62	↓ variability in patients CVR = 0.61; 95%CI: 0.46–0.80
IL-18	↑ in patients *g* = 1.97; 95%CI: 1.00–2.95	↔ CVR = 0.86; 95%CI: 0.67–1.09
sIL-1RA	↑ in patients *g* = 0.53; 95%CI: 0.18–0.89	↔ CVR = 1.00; 95%CI: 0.84–1.20
sIL-2R	↑ in patients *g* = 0.71; 95%CI: 0.44–0.98	↓ variability in patients CVR = 0.85; 95%CI: 0.73–0.99
TNF α	↑ in patients *g* = 0.54; 95%CI: 0.32–0.76	↔ CVR = 0.96; 95%CI: 0.84–1.10

## 4 Discussion

Our meta-analysis finds evidence that mean-scaled variability, measured as CVR, is reduced in patients with depression for CRP, IL-12 and sIL-2R, while it is unchanged for IL-3, IL-6, IL-18 and TNF α. In the same sample, we also find that blood levels of CRP, IL-3, IL-6, IL-12, IL-18, sIL-2R and TNF α are significantly elevated in patients with depression with medium-large effect sizes (range 0.54–1.97), and that these findings are robust to a range of potential confounds and moderators. See Table 1 for a summary of our findings.

Our study is, to our knowledge, the first meta-analysis of variability of immune parameters in individuals with depression compared to matched controls. Mean differences in inflammatory markers in depression have been meta-analysed before (Howren et al., 2009, Dowlati et al., 2010, Haapakoski et al., 2015, Goldsmith et al., 2016, Köhler et al., 2017). However, as shown in Supplementary Table 1, this study is by far the largest meta-analysis of immune markers in depression, including a sample 1.48 times larger than the largest previous one. In addition to this, this is one of the first studies to systematically consider the effect on immune markers of excluding patients not experiencing an active depressive episode (previously only considered in a much smaller study by Goldsmith et al), duration of illness (previously only considered descriptively), study quality (previously only considered in a smaller study by Haapakoski et al), and smoking (previously only considered by Kohler et al). Furthermore, our findings of increased mean levels of CRP, IL-6, IL-12 and TNF α in depression replicate previous meta-analytical findings; the same can be said of no changes in levels of TGF β (Supplementary Table 1). Reductions in IL-4, found in our study with an effect size of −0.73 and resistant to most sensitivity analyses, were not significant in Köhler et al. (2017) nor in Dowlati et al. (2010), however both these studies were based on considerably smaller samples, which could explain the difference. More controversial is the result for IFNγ, which we find not significantly altered in our main analysis and increased in patients when excluding studies not matched for smoking levels between cases and controls. Given that previous, smaller meta-analyses were also non-concordant with regards to IFNγ (Dowlati et al., 2010, Goldsmith et al., 2016, Köhler et al., 2017), we believe that more research is needed to establish the relationship between IFNγ levels and depression.

### 4.1 Interpretations and implications

#### 4.1.1 Meta-analysis of heterogeneity

In a previous study we have shown that patients with depression show a proportion of high CRP levels at different cut-offs (CRP > 1 mg/L, >3mg/L and > 10 mg/L) that is similar to matched controls (Osimo et al., 2019); this supported the hypothesis that the shape of the CRP distribution curve is similar in patients and controls. In this study we find that mean-scaled variability of CRP and of a number of other immune markers is either reduced or unchanged in patients with depression as compared to healthy controls. A reduced variability implies a narrower distribution in patients than in controls, and possibly even a greater homogeneity in the inflammatory phenotype in depression. Therefore, the findings to date, at least for markers that show elevations of the mean and reductions in heterogeneity such as CRP, support a narrower distribution that is shifted to the right in depression. This is important as in the past there have been suggestions that inflammation in depression could be due to a sub-group of “inflamed and depressed” subjects, who might potentially be part of a separate sub-group of the depressed population (Miller and Cole, 2012). Our findings, instead, point in the direction of a continuous distribution of inflammatory markers in the depressed population, which is more homogenous than the healthy population.

The reduction in variability in CRP is worthy of a special mention here, as CRP is the main inflammatory marker routinely measured in clinical practice (Yeh, 2004), and it is commonly used to stratify patients based on peripheral inflammatory levels in immunopsychiatric studies. Activation of the inflammatory system is thought to underlie antidepressant resistance (Chamberlain et al., 2018, Benedetti et al., 2002, Lanquillon et al., 2000, Carvalho et al., 2013), highlighting a potential involvement in treatment response (Carvalho et al., 2013, Maes et al., 1997, O’Brien et al., 2007, Yoshimura et al., 2009). Therefore, whether targeting inflammatory cytokines could provide therapeutic benefit for patients with depression is a key question that is being investigated in ongoing trials (e.g. NCT02473289; ISRCTN16942542). Our findings will be relevant for future studies assessing inflammation in depression, especially those recruiting patients based on their baseline inflammatory status.

#### 4.1.2 Meta-analysis of mean differences

We found increases in the average levels of type I and other pro-inflammatory cytokines such as IL-3, IL-6, IL-12, IL-18 and TNF α; we also found reductions in IL-4, one of the main anti-inflammatory and immune-modulatory cytokines; finally, we found mean increases in CRP, which is one of the best characterised inflammatory markers in medical (Danesh et al., 2000, Visser et al., 1999) and psychiatric conditions (Fernandes et al., 2016, von Känel et al., 2007, Fernandes et al., 2016). Taken together, these results confirm that acute depression is associated with a pro-inflammatory state.

CRP is one of the best studied inflammatory markers in the field of medicine. Higher levels of CRP have been consistently found in cross-sectional studies and in population-based longitudinal studies of depression, often preceding the onset of illness (Gimeno et al., 2009, Khandaker et al., 2014, Wium-Andersen et al., 2013, Zalli et al., 2016), suggesting that inflammation could be a cause rather than simply a consequence of the illness; supporting this hypothesis, recently Mendelian randomization analyses of the UK Biobank sample found that IL-6 and CRP are likely to be causally linked with depression (Khandaker et al., 2019). Furthermore, elevated peripheral CRP levels have been found to correlate with its level in the central nervous system, with a strong correlation between plasma and CSF CRP (r = 0.855, p < 0.001) (Felger et al., 2018).

TNF α is one of the major pro-inflammatory cytokines; it is produced by dendritic cells and macrophages and is a major activator of downstream inflammatory cascades with multiple effectors (Abbas et al., 2014). During acute infection dendritic cells and macrophages also produce IL-6 and IL-12; both are type I cytokine family members, secreted in response to an acute inflammatory stimulus (Abbas et al., 2014). IL-12 plays a central role in responses to active infection promoting Th1 responses and, hence, cell-mediated immunity (Stern et al., 1996). TNF α, IL-6 and IL-12 increases in current depressive episodes underline the systemic nature of the inflammatory status, showing some similarity to the immune reaction to an active infection.

For markers found to be overall not different between patients and controls, but with variable results in sensitivity analyses (IL-5, IFNγ and TGF β), our results encourage further research, aiming to disentangle their potential role in mediating effects of treatment (IL-5), smoking (IFNγ) or BMI differences (TGF β).

Finally, IL-2 and IL-8 were found to be increased in patients in our main analysis, but produced discordant results in sensitivity analyses due to the effect of BMI-matching; future studies should carefully match participants for BMI as this appears to be a particularly relevant factor affecting immune status.

### 4.2 Strengths and limitations

The main strength of this work is the use of the largest sample of studies of inflammatory markers in depression to date; the same large sample was used to study heterogeneity and mean differences in patients as compared to controls. Even if we could not make inferences on the shape of the distribution, such as modality, as this would require individual subject data, we were able to obtain the first measure to date of the variability of inflammatory markers in depression.

A further strength of this paper is the employment of a systematic approach to the analysis of potential confounds. Given the large number of studies that focussed on inflammatory markers in depression, we were able to investigate the effect of potential psychiatric (e.g. treatment status, current depressive episode at time of sampling and duration of illness) and lifestyle confounds (e.g. age, BMI and smoking status), as well as statistical and sampling confounds (e.g. data skew and study quality). Sensitivity analyses focussing on studies with strict environmental and physiological matching provided us with greater confidence that depression is associated with the elevation of some immune parameters. Use of a multivariate meta-analytic approach to reduce the influence of multiplicity is a further strength.

Among our limitations, we included cross-sectional studies which used different tools to diagnose depression, even if only studies using ICD or DSM diagnostic criteria were included. Inconsistency between studies was moderate to high. This could reflect methodological factors, e.g. differences in assay sensitivity. However, the random-effects model used is robust to inconsistency, and would not explain our variability findings, because these reflect within-study variation (with methodologic factors common to patient and control groups in any given study). Due to data unavailability, some sensitivity analyses might be subject to type II error, i.e. false negatives; for example, BMI-matched sensitivity analyses often included samples much smaller than that of the main analysis. Furthermore, sensitivity analyses of antidepressant naïve and treatment resistant patients were not possible owing to insufficient studies.

Although all studies included in analyses used well validated quantification techniques, insufficient assay sensitivity may have limited the ability to detect subtle differences in immune parameters between patients and controls, particularly for titres beneath the limit of assay detection. Unfortunately, very few studies (2 out of 106) reported the number of samples below the limit of assay detection, so this factor could not be taken into account. Positive data skew can inflate standard deviation due to outliers within the ‘tail’ of the data (Fayers, 2011). However, we demonstrated no significant difference in the proportion of skewed data sets between patients and controls, suggesting that influence of skew was equal. Thus, excessive skew in healthy controls compared with patients was not likely contributing to results.

We excluded papers that only included patients and controls presenting the same co-morbidity or physiological state in addition to depression (such as studies in autoimmune disorders or pregnancy) to reduce the risk of bias. Most included studies excluded participants with co-morbid medical conditions, and the presence of co-morbidity in participants was assessed as one of the items of our quality assessment of papers. It was not possible to exclude all co-morbidity due to original data quality, but we are confident this issue is not going to significantly affect results as a) we used random effect models to account for additional variation; b) co-morbidity is likely to be equally distributed between cases and controls; and c) our large sample (the largest to date) allows for more individual variation without affecting results.

A very limited number of studies on CRP excluded participants presenting with an acute infection (CRP > 10 mg/L); we decided to include these studies because we previously found that the odds ratio of inflammation in patients vs controls is very similar if considering all patients (OR = 1.46) or excluding patients and controls with CRP > 10 mg/L (OR = 1.44) (Osimo et al., 2019), thus suggesting that an equal proportion of patients and controls present with acute inflammation.

### 4.3 Conclusions and future directions

In this study we found a reduction in mean-scaled variability in CRP, IL-12 and sIL-2R. We found increases in the mean levels of CRP, IL-3, IL-6, IL-12, IL-18, sIL-2R and TNF α in patients with depression. These results survived sensitivity analyses for psychiatric and lifestyle predictors, influence of skew, influence of poor-quality studies and publication bias.

Our results confirm that acute depression is a pro-inflammatory state, and lend support to the hypothesis that inflammatory marker elevations in depression are not due to an inflamed sub-group, but rather to a right shift of the immune marker distribution. However, future research should specifically address the inflammatory sub-group hypothesis of depression, which can only be directly tested in an individual-patient meta-analysis.

## Conflict of interest disclosures

Professor Howes has received investigator-initiated research funding from and/or participated in advisory/speaker meetings organized by Angelini, Autifony, Heptares, Janssen, Lundbeck, Lyden-Delta, Otsuka, Servier, Sunovion, Rand, and Roche. Prof Pariante received research funding from Johnson & Johnson, the UK Medical Research Council and the Wellcome Trust; he is also part of consortia that also include Johnson & Johnson, GSK and Lundbeck. Dr Osimo, Dr Pillinger, Ms Mateos Rodriguez and Dr Khandaker report no conflicts of interest.

## Declaration of Competing Interest

The authors declare that they have no known competing financial interests or personal relationships that could have appeared to influence the work reported in this paper.
