# Supplementary material for: Inflammatory markers in depression: A meta-analysis of mean differences and variability in 5,166 patients and 5,083 controls
Source: Brain Behav Immun. 2020 Jul;87:901–9. doi: 10.1016/j.bbi.2020.02.010 (PMC7327519; doi:10.1016/j.bbi.2020.02.010)
Supplement: Supplementary data 1 [file mmc1.docx]

Osimo et al, Mean Differences and Heterogeneity of Inflammatory Markers in Depression

**SUPPLEMENTARY MATERIALS**

# SUPPLEMENTARY METHODS

## Search Strategy and Study Selection

A systematic review was performed according to PRISMA (Preferred Reporting Items for Systematic Reviews and Meta-analyses) and MOOSE guidelines. The search protocol was prospectively published on PROSPERO (<http://www.crd.york.ac.uk/PROSPERO/display_record.php?ID=CRD42018103879>). The following keywords were used: *(IL-* or cytokine or CRP or "C-reactive protein" or "hs-CRP" or hsCRP or interleukin* or "tumour necrosis factor" or "transforming growth factor" or interferon) and (depressi*)*. The search was independently performed by two study investigators (IMR and EFO) using the EndNote X8^1^.

## Selection Criteria

Abstracts were screened, and the full texts of relevant studies were retrieved.

We included cross-sectional studies that: (1) measured levels of cytokines/cytokine receptors or C-reactive protein (CRP); (2) sampled peripheral blood; (3) included patients meeting clinical criteria (DSM or ICD) for a depressive disorder; (4) included healthy controls matched to included patients. We included studies in any language. Exclusion criteria were (1) non-original or non-peer-reviewed data, e.g., reviews or conference abstracts; (2) studies where patients with depression all presented a medical or physiological condition potentially affecting inflammatory levels, e.g., myocardial infarction, cancer or pregnancy; (3) genetic studies; (4) non-human studies; (5) absence of data allowing determination of mean and/or standard deviation; (6) data were presented as already transformed with non-logarithmic transformations (e.g. square root transformation). Where insufficient data were recorded for a given immune parameter to allow for mean and/or standard deviation to be calculated in either the patient or control group, data pertaining to that parameter were excluded for both the patient and control group for that study. Where the same control group was used for different treatment groups including an untreated depression group, the treated patients were excluded. Where a sample demonstrated overlap between different papers, we excluded the smaller sample. EFO, IMR and TP applied inclusion/exclusion criteria independently and selected the final studies for this review.

## Data Extraction and Processing

Where data were available only in diagrammatic format, data were extracted using the Plot Digitizer tool ^2^. If there were multiple publications for the same data set, data were extracted from the study with the largest data set. When depression subgroups were reported, splitting patients with current depression and patients with past depression with the same controls, we only included patients experiencing a current depressive episode.

## Statistical analysis

An unstructured covariance matrix was used owing to uncertainty regarding immune parameter correlations in depression. Analyses were only performed if ≥3 studies were identified. A 2-tailed P value <.05 was deemed significant. All analyses were conducted using the *metafor* package ^3^ in the R statistical programming language ^4^.

## Meta-analysis of mean differences

The skew ratio of each immune parameter for patients and controls was determined using the following calculation: lowest possible value for each parameter subtracted from observed mean, divided by standard deviation. A ratio of <1 provides strong evidence of skew ^5,6^, and, consequently, studies with a ratio <1 were removed in sensitivity analyses. Data were log transformed before meta-analysis, since the Cochrane Collaboration recommends log transformation for normalization of positive skew ^6^, and in recognition that meta-analyses based on means are appropriate only for data that are at least approximately normally distributed ^6^. Log-transformed data were either extracted directly from manuscripts, or calculated as described by Higgins and colleagues ^6^.

## Meta-analysis of Variability

Where the mean is greater in patients than controls, the CVR is a more conservative estimate of variability. To aid interpretation, summary effect sizes for lnVR and lnCVR were transformed back to a linear scale, as previously described ^7,8^. Thus, a VR (or CVR) of 1 indicates equal variability in patient and control groups, a VR (or CVR) greater than 1 indicates greater relative variability in patient groups, and a VR (or CVR) less than 1 indicates lower relative variability in patient groups.

## Moderator and Sensitivity analyses

Study quality was assessed using the Newcastle-Ottawa Scale (NOS) ^6,9^. To determine if findings were influenced by data-skew, we conducted sensitivity analyses after excluding data sets that met Cochrane criteria for skew despite log transformation ^6^. To determine if there was a difference in proportion of skewed data between patients and controls (which could influence variability analyses), for each immune parameter, the proportion of data sets with severe skew in patients and controls was compared using Fisher’s exact test.

# SUPPLEMENTARY RESULTS

## Sensitivity analysis of influence of psychiatric clinical predictors on mean differences

Removing studies where patients were being treated with antidepressants at time of sampling created a data set of 58 studies (3,229 patients, 3,169 controls) (Supplementary Table 2 and Supplementary Figure 2). Analyses showed results concordant with the main analysis for IL-1β; IL-2, IL-4, IL-6, IL-8, IL-12, IL-13, IL-18, sIL-1RA, sIL-2R, TNFα, CRP, IFNγ and TGFβ. Results for IL-10 were no longer significant, and for IL-5 a new significant elevation not seen in the main analyses was detected. Data were not sufficient to meta-analyse IL-1α, IL-3, IL-7 and sIL-6R.

Removing studies where patients were not experiencing a current depressive episode at the time of blood sampling created a data set of 96 studies (4,644 patients, 4,569 controls) (Supplementary Table 2 and Supplementary Figure 3). Analyses produced results that are concordant with the main analysis, with the exception of a new significant elevation for IL-13 in patients with depression.

Meta-regressions of the relationship between duration of illness with cytokine levels did not produce significant results for any marker with the exception of IL-5, IL-7, IL-8 and IFNγ, in which mean duration of illness was significantly positively correlated to cytokine variation (Supplementary Table 2).

## Sensitivity analysis of influence of lifestyle and clinical variables on mean differences

Supplementary Table 3 and Supplementary Figure 4 show that age is not likely to be a significant confounding factor in our meta-analyses, as no meta regression was significant for mean patient age, and results of a meta-analysis excluding studies not matched for age (102 studies; 4,771 patients, 4,678 controls) was completely concordant with our main results.

Removing studies not matched for BMI created a data set of 56 studies (3,149 patients, 3,114 controls) (Supplementary Table 3 and Supplementary Figure 5). Results were concordant with the main analysis for IL-1β, IL-5, IL-6, IL-10, IL-12, IL-13, sIL-1RA, sIL-2R, TNFα, CRP and IFNγ. With the exception of sIL-2R and IFNγ, all these markers were not significant when meta-regressed against patient BMI (in all studies reporting this result). IL-1α, IL-7 and sIL-6R elevations became non-significant when excluding studies not matched for BMI, while TGFβ showed a new significant elevation not seen in the primary analysis. There were insufficient data for a meta-analysis of IL-3, IL-4 and IL-18. Finally, IL-2 and IL-8 showed discordant reductions in the BMI-matched sample, suggesting that BMI might have an important effect on these markers.

Removing studies not matched for smoking created a data set of 37 studies (2,586 patients, 2,587 controls) (Supplementary Table 3 and Supplementary Figure 6). Results were concordant with the main analysis for IL-5, IL-6, IL-12, IL-13, TNFα and CRP. For IL-1β, IL-2, IL-4, IL-7, IL-8, IL-10 and sIL-6R results were no longer significant in this sensitivity analysis. IFNγ showed a new significant elevation in patients. Data were insufficient to perform a meta-analysis on IL-1α, IL-3, IL-18, sIL-1RA, sIL-2R and TGFβ.

## Sensitivity analyses of the influence of skew, publication bias, sample type and study inconsistency

Removing studies with evidence of persistent severe skew despite log transformation created a data set of 82 studies (1,228 patients, 1,144 controls) (Supplementary Figure 7). Analyses showed significant elevations in the following parameters in depression, consistent with the main analysis: IL-2; IL-6; IL-10; IL-12; IL-18; IL-1Ra; IL-2R; IL-6R; TNFα; and CRP. TGFβ became significantly elevated in depression after removing one study with evidence of severe skew. Significant reductions were observed in depression in IL-4, consistent with the main analysis; a new reduction in IL-8 in depression was found. Consistent with the main analysis, no significant differences were found in IFNγ between groups, while IL-1β became non-significant.

Results of a meta-regression taking into-account assay type (ELISA vs multiplex) were identical to the main analysis.

The funnel plot for publication bias demonstrated symmetry (Supplementary Figure 8), with one outlier ^10^. Re-analysis with the outlier excluded (Supplementary Figure 9) showed that results for IL-6R were no longer significant (*g*=0.10; 95%CI: -0.12-0.32; *p*=0.39), suggesting influence of publication bias on these results. Higgins’ I^2^ inconsistency values (Supplementary Table 4) demonstrated a medium-large degree of inconsistency for all parameters.

## Variability Ratio

We found a significant overall effect of group on log variability ratio across all immune parameters (omnibus χ^2^=266.6, p<0.0001). Supplementary Figure 10 shows that the variability of depression was significantly reduced compared with controls for the following parameters: IL1β (VR=0.58; 95%CI: 0.45-0.75; *p*<0.01); IL3 (VR=0.39; 95%CI: 0.29-0.54; *p*<0.01); IL6 (VR=0.56; 95%CI: 0.46-0.67; *p*<0.01); IL12 (VR=0.45; 95%CI: 0.28-0.73; *p*<0.01); IL13 (VR=0.32; 95%CI: 0.17-0.58; *p*<0.01); IL18 (VR=0.55; 95%CI: 0.40-0.76; *p*<0.01); IL1RA (VR=0.66; 95%CI: 0.49-0.88; *p=*0.01); IL2R (VR=0.74; 95%CI: 0.62-0.88; *p<*0.01); TNFα (VR=0.74; 95%CI: 0.61-0.90; *p<*0.01); CRP (VR=0.56; 95%CI: 0.46-0.70; *p<*0.01); and IFNγ (VR=0.58; 95%CI: 0.43-0.80; *p<*0.01). Variability was not significantly altered for: IL1α (VR=0.70; 95%CI: 0.47-1.05; *p=*0.09); IL2 (VR=0.76; 95%CI: 0.58-1.00; *p=*0.05); IL4 (VR=1.08; 95%CI: 0.80-1.46; *p=*0.62); IL5 (VR=0.87; 95%CI: 0.70-1.08; *p=*0.20); IL7 (VR=1.06; 95%CI: 0.89-1.28; *p=*0.50); IL8 (VR=0.90; 95%CI: 0.53-1.53; *p=*0.69); IL10 (VR=0.74; 95%CI: 0.47-1.15; *p=*0.18); IL6R (VR=0.87; 95%CI: 0.70-1.08; *p=*0.21); and TGFβ (VR=0.85; 95%CI: 0.85-1.28; *p=*0.71). Excluding poor quality studies, results of the VR meta-analyses for IL12 and IL18 became non-significant, while IL2 showed greater variability in controls, with implications for reliability of the main outcome (Supplementary Figure 13).

# SUPPLEMENTARY FIGURES

## Supplementary Figure 1: PRISMA diagram

_
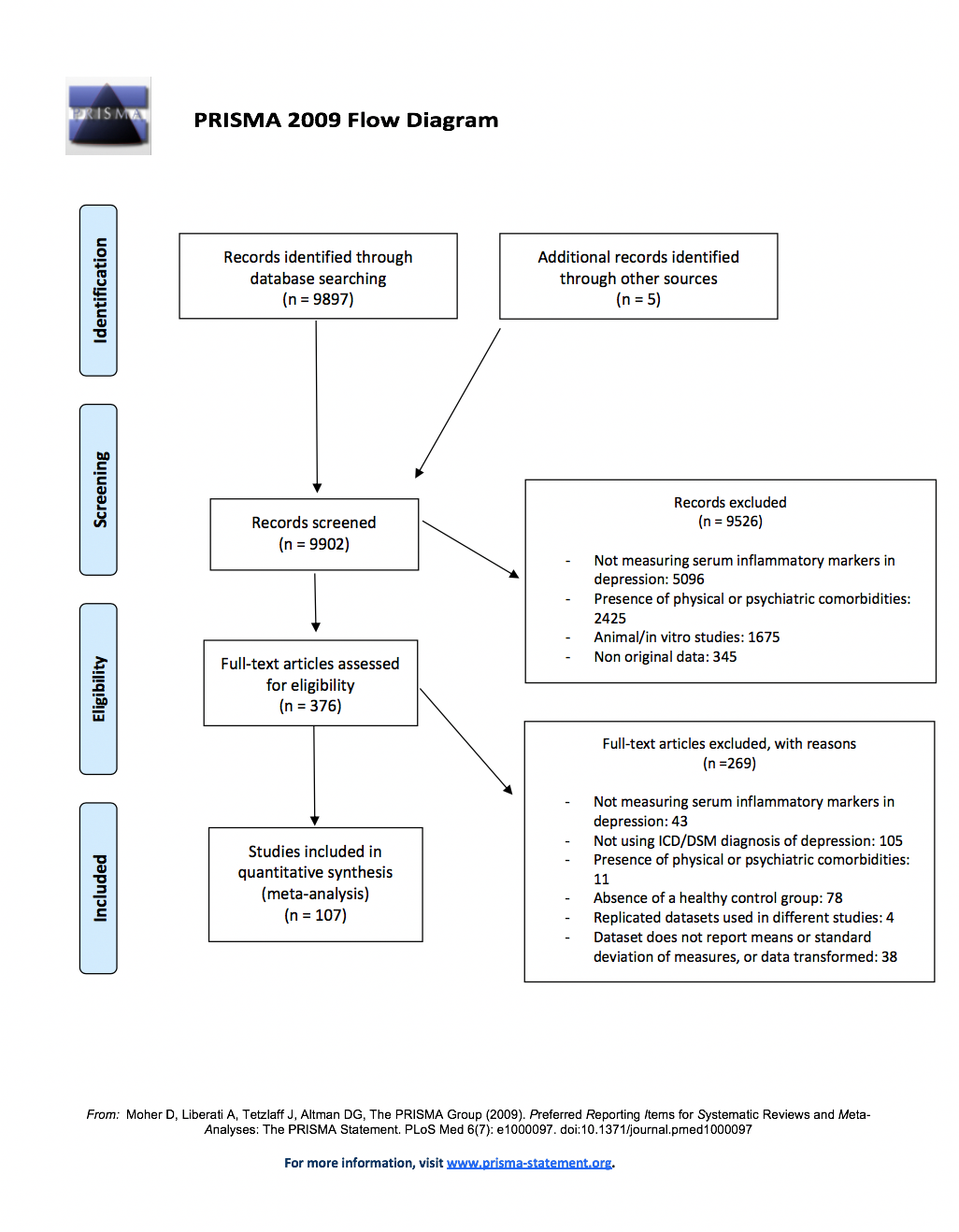
_

## Supplementary Figure 2: Forest plot showing effect sizes for mean differences in log transformed immune parameters in depression compared with healthy controls – only studies including untreated patients.


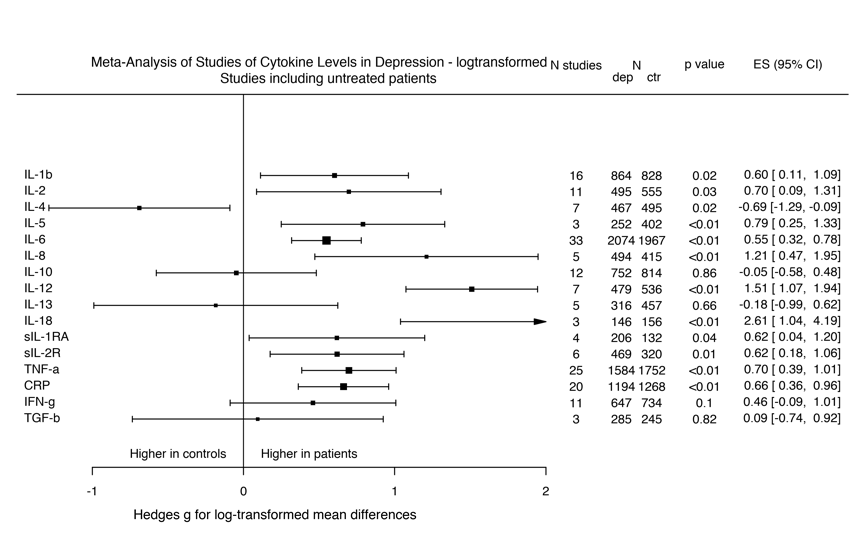


## Supplementary Figure 3: Forest plot showing effect sizes for mean differences in log transformed immune parameters in depression compared with healthy controls – only including studies of a current depressive episode


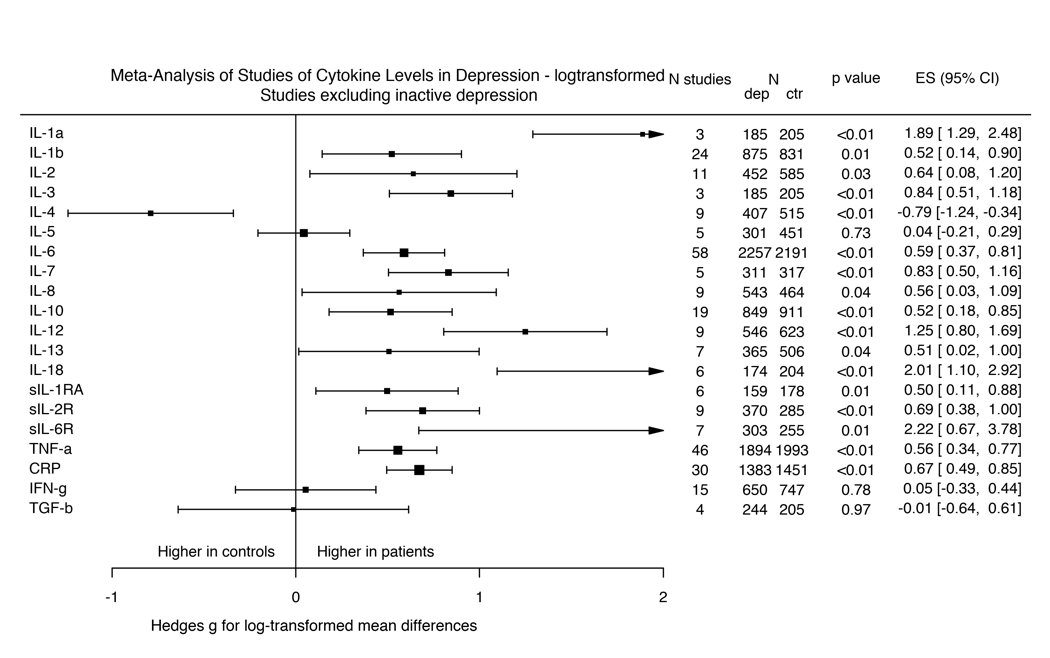


## Supplementary Figure 4: Forest plot showing effect sizes for mean differences in immune parameters in depression compared with healthy controls – excluding studies not matched for age.


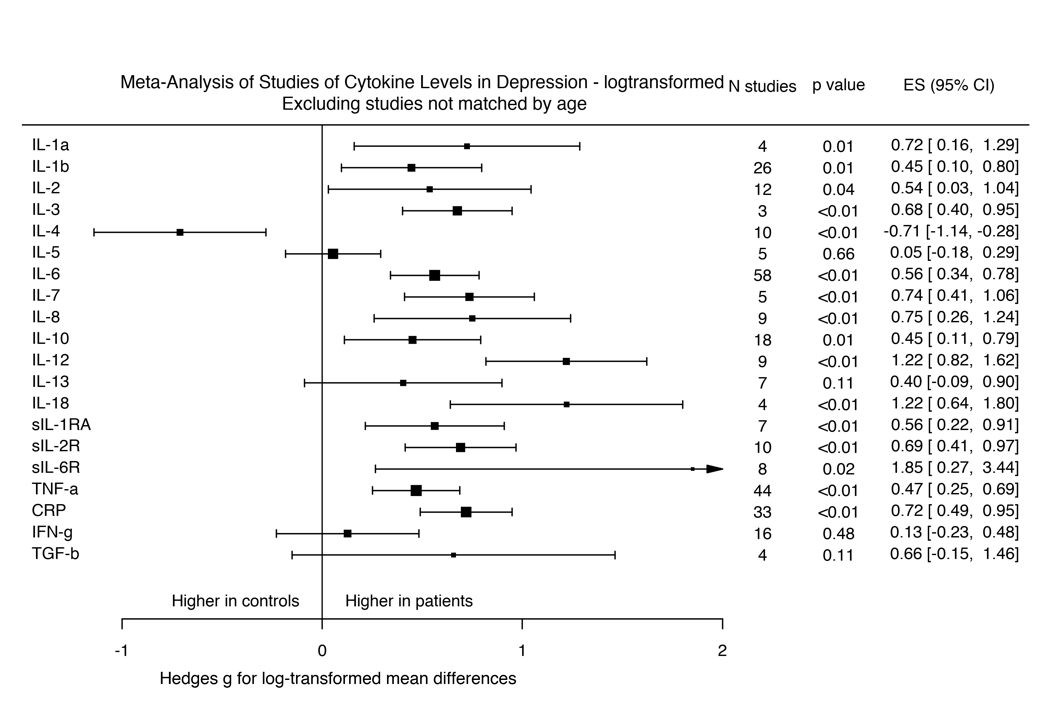


## Supplementary Figure 5: Forest plot showing effect sizes for mean differences in immune parameters in depression compared with healthy controls – excluding studies not matched for BMI.


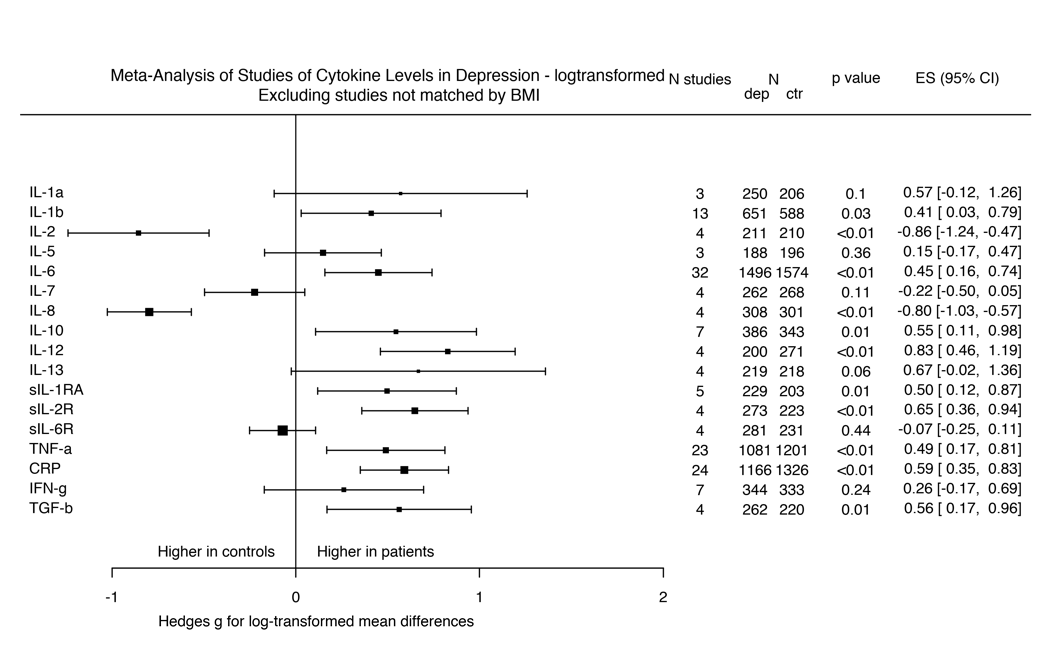


## Supplementary Figure 6: Forest plot showing effect sizes for mean differences in immune parameters in depression compared with healthy controls – excluding studies not matched for smoking.


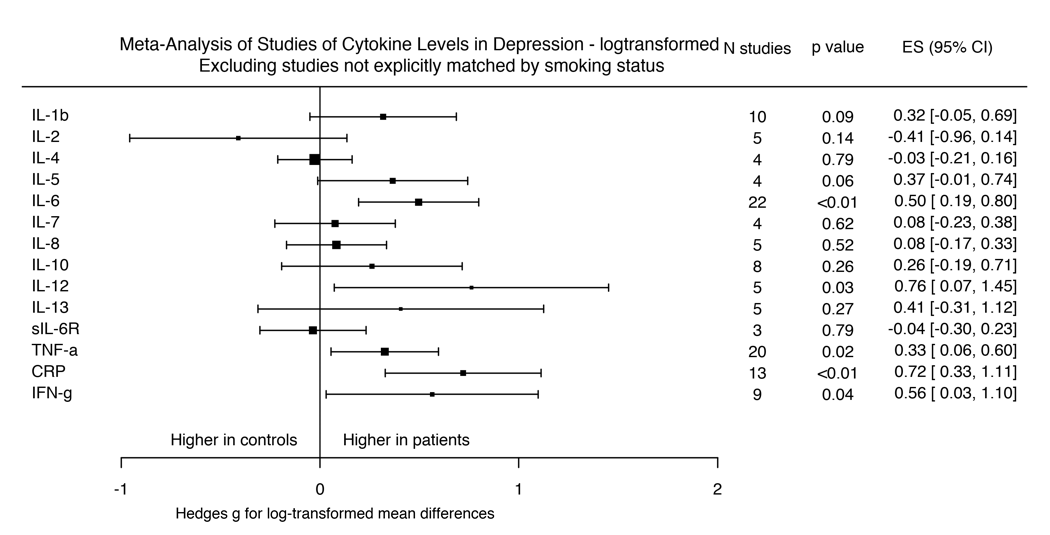


## Supplementary Figure 7: Forest plot showing effect sizes for mean differences in log transformed immune parameters in depression compared with healthy controls – non-skewed dataset.


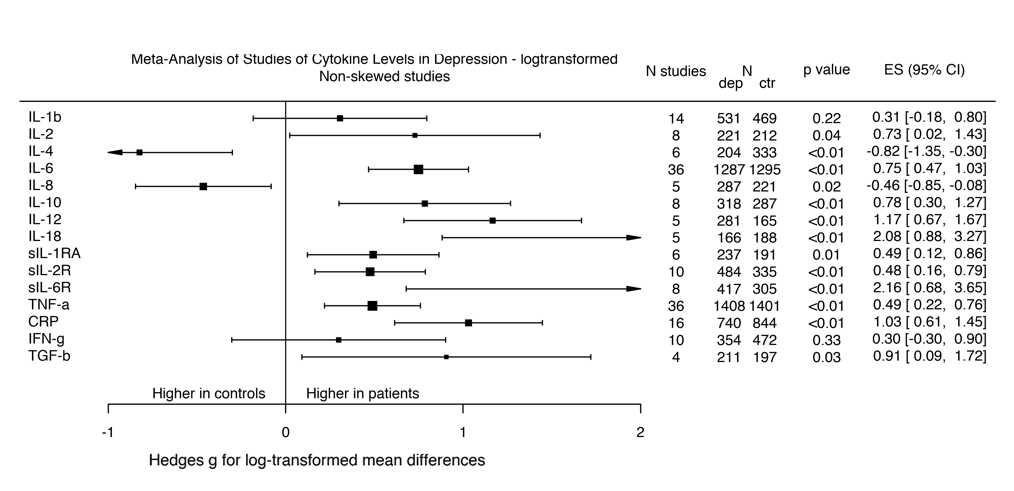


## Supplementary Figure 8: Funnel plot of immune parameters in depression.

Outlier in red

**
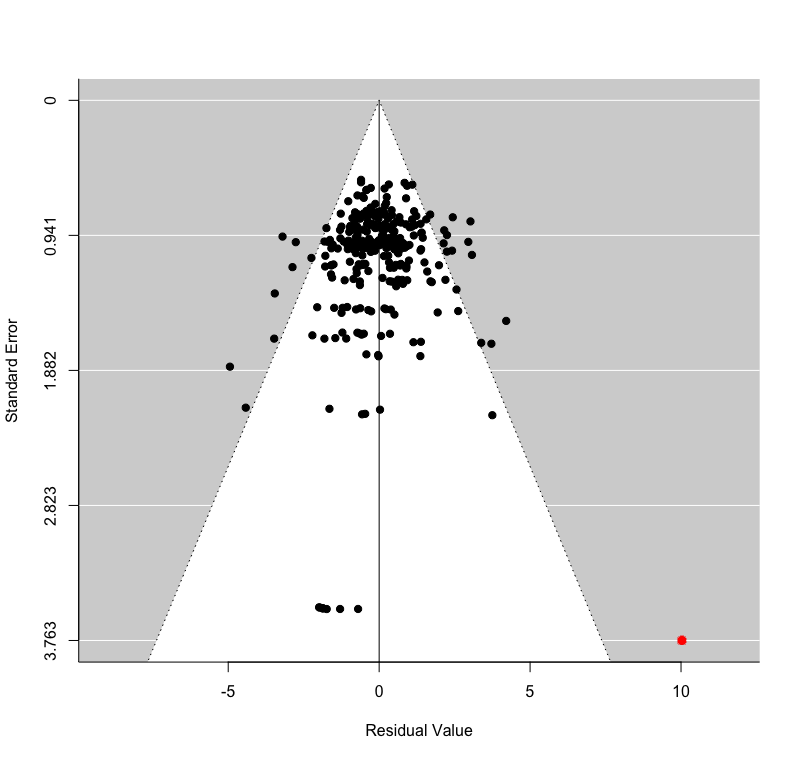
**

## Supplementary Figure 9: Forest plot showing effect sizes for mean differences in log transformed immune parameters in depression compared with healthy controls – excluding one outlier (Camardese et al, 2011).


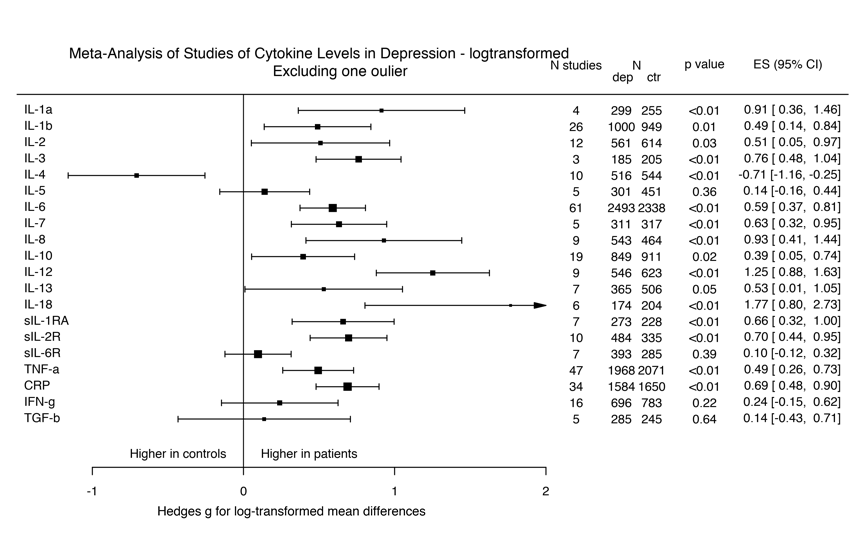


Supplementary Figure 10: Forest plot showing effect sizes for variability ratio (VR) of immune parameters in depression compared with healthy controls.

*
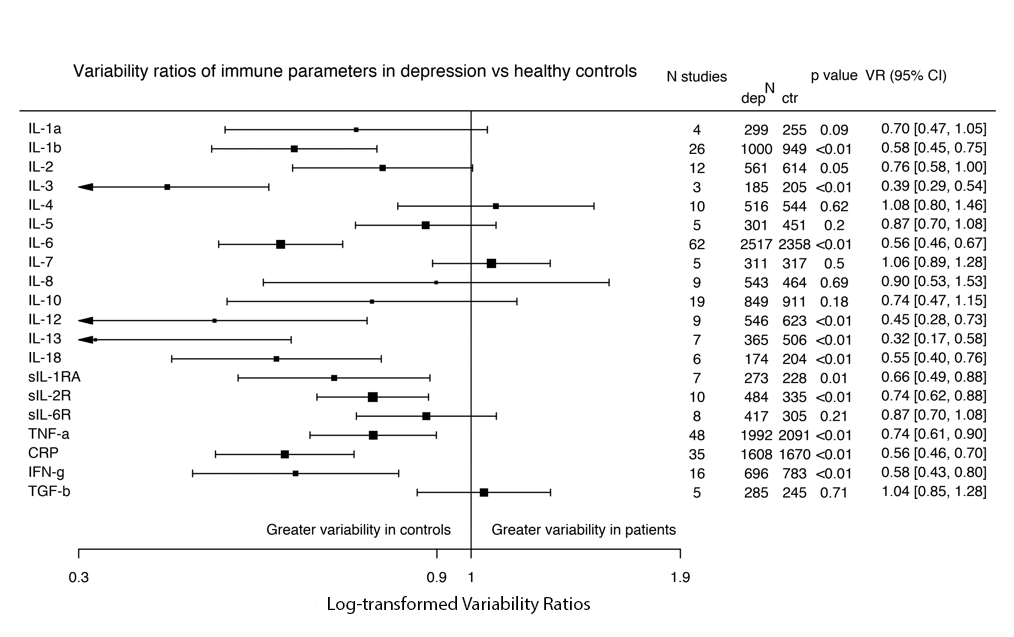
*

## Supplementary Figure 11: Forest plot showing effect sizes for mean differences in log transformed immune parameters in depression compared with healthy controls – excluding poor quality studies.


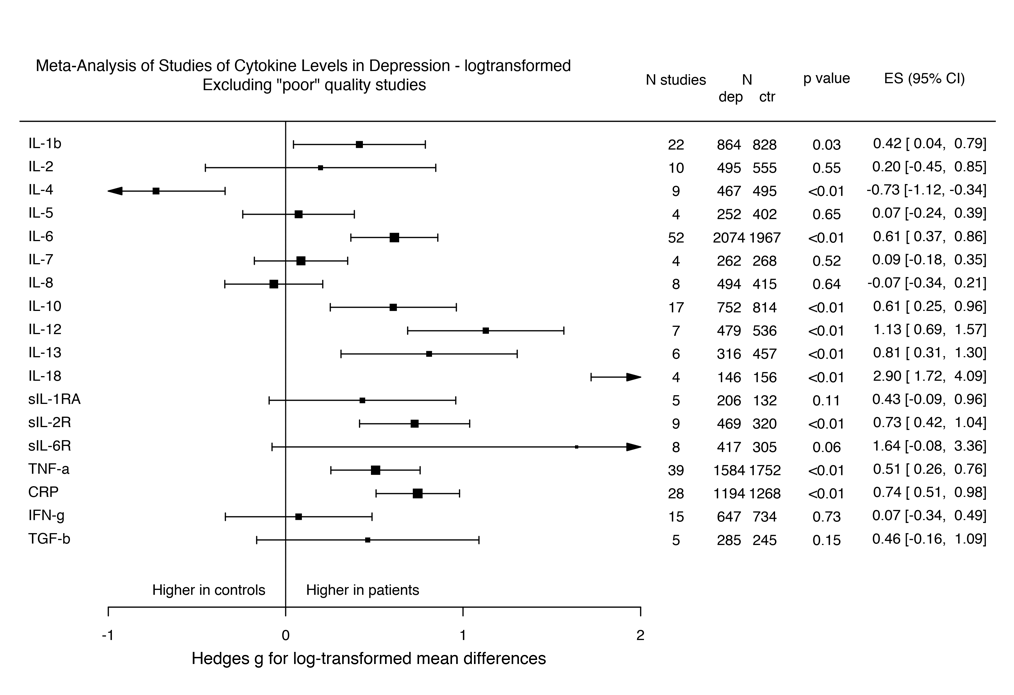


## Supplementary Figure 12: Forest plot showing effect sizes for Coefficient of variation ratio (CVR) of immune parameters in depression compared with healthy controls – excluding poor quality studies.


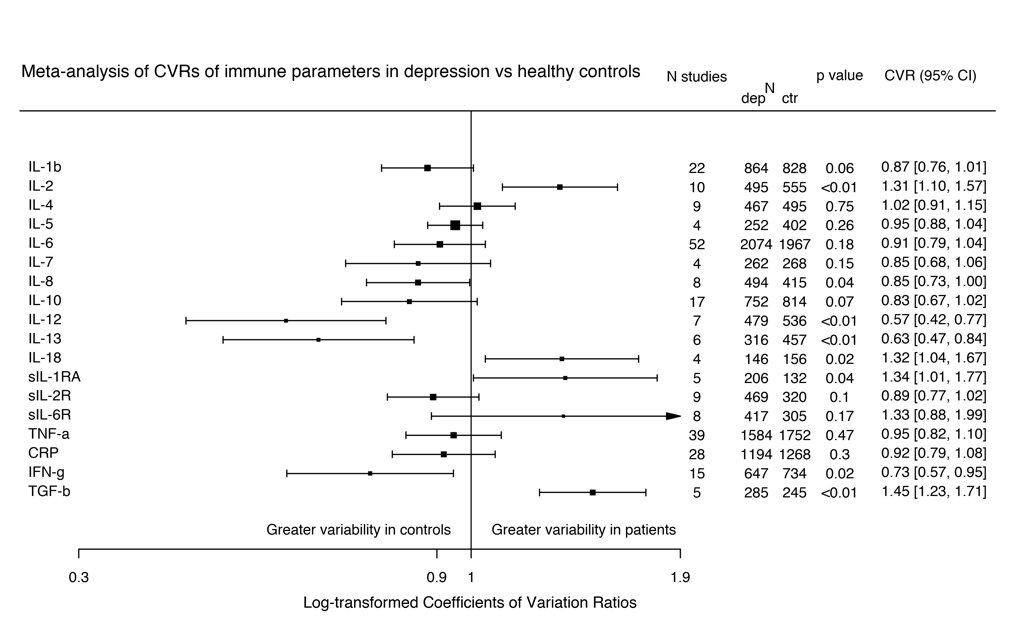


## Supplementary Figure 13: Forest plot showing effect sizes for variability ratio (VR) of immune parameters in depression compared with healthy controls – excluding poor quality studies.


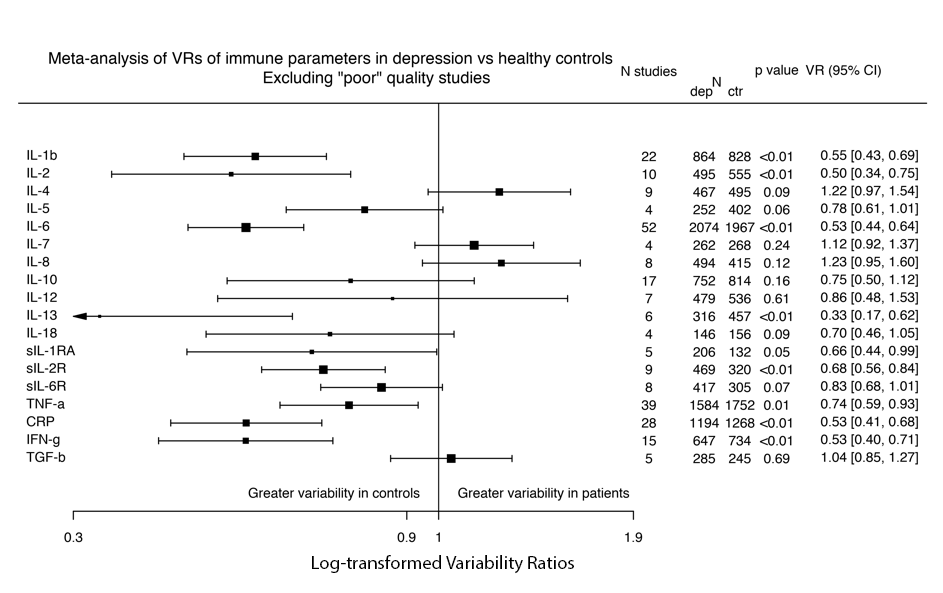


# SUPPLEMENTARY TABLES

### Supplementary Table 1: Summary of Meta-Analytic Findings in Depression

✓ indicates that the factor was available/considered in the study, while 🗶 indicates that the factor was not available/not considered.

|  | Osimo et al, 2019 | Köhler et al, 2017 | Goldsmith et al, 2016 | Haapakoski, 2015 | Dowlati et al, 2010 | Howren et al, 2009 |
| --- | --- | --- | --- | --- | --- | --- |
| Number of studies | 107 | 82 | 30 | 58 | 24 | 51 |
| Number of patients | 5166 | 3212 | 2568 | 3496 | 2011 | 🗶 |
| Number of immune markers | 20 | 20 | 11 | 4 | 8 | 3 |
| Meta-analysis of variability | ✓ | 🗶 | 🗶 | 🗶 | 🗶 | 🗶 |
| Mean meta-analysis results in depression for: | | | | | | |
| CRP | ↑ in patients | 🗶 | 🗶 | ↑ in patients | 🗶 | ↑ in patients |
| IL-6 | ↑ in patients | ↑ in patients | ↑ in patients | ↑ in patients | ↑ in patients | ↑ in patients |
| IL-4 | ↓ in patients | ↔ (smaller sample) | ↓ in patients | 🗶 | ↔ (smaller sample) | 🗶 |
| IL-12 | ↑ in patients | ↑ in patients | ↑ in patients | 🗶 | 🗶 | 🗶 |
| TNFα | ↑ in patients | ↑ in patients | ↑ in patients | ↑ in patients | ↑ in patients | 🗶 |
| IFNγ | ↔ | ↓ in patients | ↓ in patients | 🗶 | ↔ | 🗶 |
| TGFβ | ↔ | ↔ | 🗶 | 🗶 | 🗶 | 🗶 |
| Adjustment/consideration of: | | | | | | |
| Effect of antidepressants | ✓ | Only descriptive | ✓ | ✓ | Only on antidepressant-free | ✓ |
| Current depressive episode | ✓ | 🗶 | ✓ | 🗶 | 🗶 | 🗶 |
| Duration of illness | ✓ | Only descriptive | Only for 2 cytokines | 🗶 | 🗶 | 🗶 |
| Study quality | ✓ | Only descriptive | 🗶 | ✓ | 🗶 | 🗶 |
| Age | ✓ | ✓ | Only for 2 cytokines | ✓ | 🗶 | ✓ |
| BMI | ✓ | ✓ | Only for 2 cytokines | ✓ | 🗶 | ✓ |
| Smoking | ✓ | ✓ | Only for 2 cytokines | 🗶 | 🗶 | 🗶 |

## Supplementary Table 2: Studies examining immune parameters in depression, meeting inclusion criteria. All studies used case-control designs.

| **Study** | **Patient N** | **Control N** | **Diagnoses** | **Patient age, mean** | **Immune Parameter** | **Matching** |
| --- | --- | --- | --- | --- | --- | --- |
| Alcocer-Gomez et al., 2014 | 40 | 20 | Major depressive disorder | 54 | IL-1b, IL-18 | Age, gender, BMI |
| Alesci et al., 2005 | 9 | 9 | Major depressive disorder | 34.7 | IL-6 | Age, gender, BMI, smoking, |
| Ali et al., 2017 | 40 | 40 | Major depressive disorder | 64.13 | IL-6 | Age, gender, BMI |
| Bai et al., 2014 | 109 | 126 | Major depressive disorder | 41.96 | CRP, IL-2R, IL-6R | Age, gender, BMI |
| Basterzi et al., 2005 | 23 | 23 | Major depressive disorder | 33.8 | IL-6 | Age, gender, smoking, |
| Berk et al., 1997 | 28 | 21 | Major depressive disorder | NA | IL-6, CRP | Age, gender, smoking, |
| Boettger et al., 2010 | 15 | 15 | Major depressive disorder | 40.7 | IL-10 | Age, gender, BMI |
| Brambilla et al., 1998 | 10 | 10 | Major depressive disorder | 72 | IL-1B, IL-6, TNF-a | Age, gender |
| Camardese, 2011 | 24 | 20 | Major depressive disorder | 46.8 | CRP | Age, gender |
| Cassano et al., 2017 | 118 | 118 | Major depressive disorder | 42.1 | IFN-g, IL-1a, IL-1b, IL-2, IL-3, IL-4, IL-5, IL-6, IL-7, IL-8, IL-10, IL-12, IL-13, TNF-a | Age, gender, BMI, smoking, |
| Chamberlain et al., 2018 | 48 | 54 | Major depressive disorder | 35.1 | CRP | Age, gender, BMI, smoking, |
| Charlton et al., 2018 | 34 | 34 | Major depressive disorder | 67.21 | IL-1b, TNF-a, IL-6 | Age, gender, BMI, smoking |
| Chavda et al., 2011 | 98 | 100 | Major depressive disorder | NA | CRP | Age, gender |
| Chen et al., 2017 | 25 | 20 | Major depressive disorder | NA | IL-2, IL-4, IL-6, IL-10, TNF-a, IFN-g | Age, gender |
| Crnkovic et al., 2012 | 38 | 36 | Major depressive disorder | NA | CRP, IL-6, TNF-a | Gender |
| Dannehl et al., 2014 | 41 | 45 | Major depressive disorder | 33.2 | TNF-a, IL-6 | Age, gender, BMI, smoking |
| Davami et al., 2016 | 41 | 40 | Major depressive disorder | 35.6 | TGF-b | Age, gender, BMI, smoking |
| Dhabhar et al., 2009 | 12 | 11 | Major depressive disorder | 38.42 | IL-10, IL-6 | Age, gender |
| Dinan et al., 2009 | 34 | 24 | Major depressive disorder | NA | TNF-a, IL-6 | Gender |
| Diniz et al., 2010 | 28 | 39 | Major depressive disorder | 70.4 | TNF-a, IL-1b | Age, gender, |
| Dome et al., 2009 | 33 | 16 | Major depressive disorder | 40.6 | CRP, TNF-a | Age, gender, BMI, smoking |
| Dunjic-Kostic et al., 2013 | 47 | 39 | Major depressive disorder | 51 | IL-6, TNF-a | Age, gender, BMI, smoking |
| Elderkin-Thompson et al., 2012 | 42 | 45 | Major depressive disorder | 69.7 | IL-6, CRP | Age, gender, BMI |
| Eller et al., 2008 | 100 | 45 | Major depressive disorder | 31.5 | IL-2r, IL-8, TNF-a | Age, gender |
| Elomaa et al., 2012 | 58 | 58 | Major depressive disorder | 53.4 | IL-5, IL-13, IFN-g | Age, gender, BMI, smoking |
| Euteneuer et al., 2012 | 25 | 22 | Major depressive disorder | 31.47 | IL-2r | Age, gender, BMI, smoking |
| Euteneuer et al., 2011 | 37 | 48 | Major depressive disorder | 33.46 | IL-6, TNF-a | Age, gender, BMI |
| Fan et al., 2017 | 64 | 80 | Depressive disorder | 35.17 | TNF-a, IL-6, IL-18 | Age, gender |
| Fornaro et al., 2011 | 16 | 16 | Major depressive disorder | 51.1 | IL-6 | Age, gender |
| Fornaro et al., 2013 | 30 | 32 | Major depressive disorder | 48.27 | IL-1b, IL-2, IL-4, IL-10, IL-12, IFN-g, TNF-a | Age, gender, smoking |
| Frodl et al., 2012 | 40 | 43 | Major depressive disorder | 41.4 | CRP, IL-6 | Age, gender, BMI |
| Frommberger, 1997 | 4 | 12 | Major depressive disorder | 46 | IL-6 | Age, gender |
| Gazal et al., 2015 | 82 | 94 | Major depressive disorder | 21.7 | IL-10 | Age |
| Goyal et al., 2018 | 50 | 50 | Depressive disorder | 37.22 | IL-2, IL-6, CRP | Age, gender, BMI |
| Grosse et al., 2016 | 71 | 69 | Major depressive disorder | 33 | IL-6, IL-7 | Age, gender, BMI, smoking |
| Hafner et al., 2008 | 70 | 68 | Major depressive disorder | 49.3 | CRP | Age, gender, BMI |
| Hernandez et al., 2008 | 31 | 22 | Major depressive disorder | 32 | IL-2, IFN-g, IL-13, IL-10, IL-1b | Age, gender, BMI, smoking |
| Ho et al., 2017 | 12 | 20 | Major depressive disorder | 23.2 | IL-1b, IL-1RA, IL-2, IL-4, IL-5, IL-6, IL-7, IL-10, IL-13, IFN-g, TNF-a, IL-8 | Age, gender, BMI, smoking |
| Hocaoglu et al., 2012 | 30 | 30 | Major depressive disorder | 38 | IL-1b, IL-6, IL-8, IL-10, IFN.g, TNF-a | Age, gender, smoking |
| Hosseini et al., 2007 | 36 | 15 | Major depressive disorder | 39.1 | IL-4, IL-10, TNF-a, IFN-g | Age, gender |
| Huang et al., 2007 | 23 | 31 | Major depressive disorder | 35.7 | CRP | Age, gender, BMI |
| Hughes et al., 2012 | 39 | 39 | Major depressive disorder | 41.9 | IL-1b, TNF-a, IL-6, IFN-g, CRP | Age, gender, BMI, smoking |
| Hung, 2007 | 21 | 14 | Major depressive disorder | 22.9 | CRP, TNF-a, IL-6 | Age, gender, BMI, smoking |
| Jozuga et al., 2003 | 17 | 10 | Major depressive disorder | 40.3 | IL-2 | Age, gender |
| Kagaya et al., 2001 | 9 | 12 | Major depressive disorder | 31.1 | IL-1b, IL-6, IL-2R, TNF-a | Age, gender |
| Kageyama et al., 2017 | 109 | 29 | Major depressive disorder | 46 | IL-6, IL-2, IL-4 | Age, gender |
| Kahl et al., 2015 | 27 | 19 | Major depressive disorder | 43.4 | IL-6, TNF-a | Age, gender, BMI, smoking |
| Karlovic et al., 2012 | 55 | 18 | Major depressive disorder | 49.6 | CRP, IL-6, TNF-a | Age, gender, smoking |
| Keri et al., 2014 | 50 | 30 | Major depressive disorder | 22.6 | CRP, IL-6 | Age, gender, BMI, smoking |
| Kim et al., 2002 | 34 | 85 | Major depressive disorder | 39.6 | IL-12 | Age, gender |
| Kling et al., 2007 | 18 | 18 | Major depressive disorder | 41 | CRP | Age, gender |
| Kokai et al., 2002 | 8 | 16 | Major depressive disorder | 37.6 | IL-18 | Age, gender |
| Kubera et al., 2000 | 9 | 10 | Major depressive disorder | 47.3 | IL-6, IL-10, IL-1RA | Age, gender |
| Lanquillon et al., 2000 | 24 | 15 | Major depressive disorder | 53.5 | CRP | Age, gender |
| Lee et al., 2006 | 30 | 30 | Major depressive disorder | 37.63 | IL-12, TGF-b | Age, gender, BMI |
| Lee et al., 2009 | 18 | 38 | Major depressive disorder | 71.4 | IL-1a, IL-1RA, IL-3, IL-12, IL-18 | Age, gender, BMI |
| Lehto et al., 2010 | 61 | 61 | Major depressive disorder | 53.74 | IL-7, CRP, IL-8 | Age, gender, BMI, smoking |
| Leo et al., 2006 | 46 | 46 | Major depressive disorder | 34.85 | IL-1b, IL-6, TNF-a | Age, gender, BMI |
| Maes et al., 1997 | 35 | 15 | Major depressive disorder | 50.3 | IL-1RA, IL-6r, IL-6 | Age, gender |
| Maes et al., 1990 | 10 | 10 | Major depressive disorder | 44.1 | IL-2r | Age, gender |
| Manoharan et al., 2016 | 73 | 44 | Major depressive disorder | 36.1 | IL-6 | Age, gender, BMI, smoking |
| Marques-Deak et al., 2007 | 45 | 38 | Major depressive disorder | 39.4 | IL-1b, IL-6, IFN-g | Age, gender, BMI, smoking |
| Merendino et al., 2002 | 10 | 10 | Major depressive disorder | 38.6 | IL-18 | Age, gender |
| Mikova et al., 2001 | 28 | 15 | Major depressive disorder | 47.3 | IL-8, IL-6, TNF-a, sIL-2R | Age, gender |
| Miller et al., 2005 | 36 | 36 | Major depressive disorder | 26.6 | CRP, IL-6, TNF-a | Age, gender, BMI, smoking |
| Mota et al., 2013 | 80 | 80 | Major depressive disorder | 26.63 | IL-1b | Age, gender, BMI, smoking |
| Motivala et al., 2005 | 22 | 18 | Major depressive disorder | 44.4 | IL-6, IL-6R | Age, gender, BMI, smoking |
| Munjiza et al., 2018 | 64 | 53 | Major depressive disorder | 45.98 | IL-6 | Age, gender |
| Narita et al., 2006 | 21 | 20 | Major depressive disorder | 60.9 | TNF-a | Age, gender, BMI |
| Nunes et al., 2012 | 77 | 78 | Depressive disorder | 47.2 | IL-6, TNF-a, CRP | Age, gender, BMI, smoking |
| O’Brien et al., 2007 | 28 | 24 | Major depressive disorder | 44.15 | IL-6, IL-8, TNF-a, IL-6R, IL-10 | Age, gender, smoking |
| O’Brien et al., 2006 | 32 | 20 | Major depressive disorder | 44 | CRP | Age, gender |
| O’Donovan et al., 2013 | 74 | 48 | Major depressive disorder | 51.3 | TNF-a, IL-6, IL-10, CRP, TGF-b | Gender, BMI |
| Oglodek et al., 2018 | 180 | 40 | Major depressive disorder | NA | IL-12 | Age, gender |
| Owen et al., 2008 | 20 | 20 | Major depressive disorder | NA | IL-1b | Age, gender |
| Pavon et al., 2006 | 33 | 33 | Major depressive disorder | 33.6 | IL-1b, TNF-a, IL-6, IL-2, IFN-g, IL-13 | Age, gender |
| Pike et al., 2006 | 25 | 25 | Major depressive disorder | 42.5 | IL-2r | Age, gender, BMI |
| Piletz et al., 2009 | 22 | 17 | Major depressive disorder | 39.4 | TNF-a, IL-1b, CRP | Age, gender, BMI, smoking |
| Rapaport et al., 1996 | 15 | 15 | Major depressive disorder | 44.6 | IL-2r | Age, gender |
| Rawdin et al., 2013 | 20 | 18 | Major depressive disorder | 37 | IL-6, IL-10 | Age, gender, BMI, smoking |
| Rief et al., 2001 | 36 | 37 | Major depressive disorder | 42.25 | IL-6, IL-1ra, IL-6r | Age, gender, BMI |
| Rivazi et al., 2016 | 31 | 30 | Major depressive disorder | 34.3 | TNF-a, IL-1b, IL-6 | Age, gender |
| Rudolf et al., 2014 | 32 | 24 | Major depressive disorder | 34.82 | IL-6, CRP | Age, gender, BMI |
| Rudzki et al., 2017 | 34 | 29 | Major depressive disorder | 42.26 | TNF-a, IL-6 | Age, gender, BMI |
| Rybka et al., 2013 | 15 | 19 | Depressive disorder | 59.7 | TNF-a | Age, gender, BMI |
| Savitz et al., 2015 | 49 | 58 | Major depressive disorder | 35.4 | CRP, IL-1ra | Age, gender, BMI |
| Schlatter et al., 2004 | 10 | 15 | Major depressive disorder | 43.8 | IL-1b, IL-6, TNF-a, | Age, gender |
| Schmidt et al., 2014 | 64 | 206 | Major depressive disorder | 39.09 | IL-2, IL-4, IL-5, IL-10, IL-12, IL-13, IFN-g, TNF-a | Age, gender, BMI, smoking |
| Seidel et al., 1995 | 39 | 39 | Major depressive disorder | 39.9 | CRP | Age, gender |
| Shen et al., 2010 | 34 | 40 | Major depressive disorder | 42.54 | IL-18 | Age, gender |
| Simon et al., 2008 | 49 | 49 | Major depressive disorder | 41.65 | IL-1a, IL-1b, IL-2, IL-3, IL-4, IL-5, IL-6, IL-7, IL-8, IL-10, IL-12, IL-13, IFN-g, TNF-a | Age, gender |
| Sluzewska et al., 1996 | 49 | 15 | Major depressive disorder | 42.3 | IL-6, sIL-6r, sIL-2r, CRP | Age, gender |
| Sowa-Kucma et al., 2018 | 114 | 50 | Major depressive disorder | 49.4 | sIL-1ra, IL-1a, sIL-2r, sIL-6r | Age, gender, BMI, smoking |
| Sugimoto et al., 2018 | 35 | 53 | Major depressive disorder | 46.3 | IL-1b, IL-6, IFN-g, TNF-a | Age, gender |
| Sutcigil et al., 2007 | 23 | 25 | Major depressive disorder | 34.78 | IL-2, IL-4, IL-12, TNF-a, TGF-b | Age, gender |
| Thomas et al., 2005 | 19 | 21 | Major depressive disorder | 76.4 | IL-1b, CRP | Age, gender |
| Tuglu et al., 2003 | 26 | 17 | Major depressive disorder | 39.38 | CRP, TNF-a | Age, gender, smoking |
| Vaccarino et al., 2008 | 81 | 275 | Major depressive disorder | 53.6 | IL-6, CRP, TNF-a | Age, gender, BMI |
| Wang et al., 2018 | 60 | 60 | Depressive disorder | 45.09 | CRP | Age, gender, BMI |
| Wiener et al., 2017 | 48 | 48 | Major depressive disorder | 21.81 | IL-6, IL-10 | Age, gender |
| Xia et al., 2018 | 76 | 76 | Major depressive disorder | 35.53 | IL-6, CRP, TNF-a | Age, gender, BMI |
| Yang et al., 2007 | 33 | 23 | Major depressive disorder | 42.12 | IL-6, IL-1b, TNF-a | Age, gender, BMI |
| Yoshimura et al., 2009 | 51 | 30 | Major depressive disorder | 39.8 | IL-6, TNF-a | Age, gender, smoking |
| Yoshimura et al., 2010 | 20 | 20 | Major depressive disorder | 39.2 | IL-6 | Age, gender |
| Zincir et al., 2016 | 50 | 30 | Major depressive disorder | 33 | IL-1b, TNF-a, IL-10, IL-4, IFN-g | Age, gender |
| Zoga et al., 2014 | 40 | 40 | Major depressive disorder | 51.1 | CRP, IFN-g, TNF-a | Age, gender, BMI |
| Zou et al., 2018 | 117 | 102 | Major depressive disorder | 37 | IL-1b, IL-6, IL-10, IL-8, TNF-a, TGF-b | Age, gender, BMI |

## Supplementary Table 3: Sensitivity Analyses of Psychiatric Predictors for mean differences in immune parameters in depression compared with healthy controls.

Results in bold are significant/significantly different from the main analysis.

| **cytokine** | **UNTREATED**  **sensitivity analysis excluding studies with treated patients** | | **CURRENT DEPRESSIVE EPISODE**  **sensitivity analysis excluding studies with patients with history of depression** | | **DURATION OF ILLNESS**  **Meta-regression with duration of illness** | |
| --- | --- | --- | --- | --- | --- | --- |
|  | **Result**  ***g*,** 95%CI, *p* | **Concordance with main analysis** | **Result**  ***g*,** 95%CI, *p* | **Concordance with main analysis** | **N of studies** | **Test of Moderators for duration of illness (months)** |
| **IL-1α** | *N/A* | *N/A* | *g*=1.89; 95%CI: 1.29-2.48; *p*<0.01 | concordant  ↑ in patients | *N/A* | *N/A* |
| **IL-1β** | *g*=0.60; 95%CI: 0.11-1.09; *p*=0.02 | concordant  ↑ in patients | *g*=0.52; 95%CI: 0.14-0.90; *p*=0.01 | concordant  ↑ in patients | 8 | QM(df = 1) = 0.0122, p-val = 0.9121 |
| **IL-2** | *g*=0.70; 95%CI: 0.09-1.31; *p*=0.03 | concordant  ↑ in patients | *g*=0.64; 95%CI: 0.08-1.20; *p*=0.03 | concordant  ↑ in patients | 5 | QM(df = 1) = 0.0219, p-val = 0.8823 |
| **IL-3** | *N/A* | *N/A* | *g*=0.84; 95%CI: 0.51-1.18; *p*<0.01 | concordant  ↑ in patients | *N/A* | *N/A* |
| **IL-4** | *g*=-0.69; 95%CI: -1.29- -0.09; *p=*0.02 | concordant  ↓ in patients | *g*=-0.79; 95%CI: -1.24- -0.34; *p<*0.01 | concordant  ↓ in patients | 6 | QM(df = 1) = 2.7669, p-val = 0.0962 |
| **IL-5** | *g*=0.79; 95%CI: 0.25-1.33; *p*<0.01 | **↑ in patients** | *g*=0.04; 95%CI: -0.21-0.29; *p*=0.73 | concordant  ↔ in patients | 4 | **QM(df = 1) = 9.2321, p-val = 0.0024** |
| **IL-6** | *g*=0.55; 95%CI: 0.32-0.78; *p*<0.01 | concordant  ↑ in patients | *g*=0.59; 95%CI: 0.37-0.81; *p*<0.01 | concordant  ↑ in patients | 18 | QM(df = 1) = 1.8298, p-val = 0.1761 |
| **IL-7** | *N/A* | *N/A* | *g*=0.83; 95%CI: 0.50-1.16; *p*<0.01 | concordant  ↑ in patients | 3 | **QM(df = 1) = 17.3059, p-val < .0001** |
| **IL-8** | *g*=1.21; 95%CI: 0.47-1.95; *p*<0.01 | concordant  ↑ in patients | *g*=0.56; 95%CI: 0.03-1.09; *p*=0.04 | concordant  ↑ in patients | 5 | **QM(df = 1) = 31.2052, p-val < .0001** |
| **IL-10** | *g*=-0.05; 95%CI: -0.58-0.48; *p*=0.86 | not significant | *g*=0.52; 95%CI: 0.18-0.85; *p*<0.01 | concordant  ↑ in patients | 8 | QM(df = 1) = 1.3432, p-val = 0.2465 |
| **IL-12** | *g*=1.51; 95%CI: 1.07-1.94; *p*<0.01 | concordant  ↑ in patients | *g*=1.25; 95%CI: 0.80-1.69; *p*<0.01 | concordant  ↑ in patients | 6 | QM(df = 1) = 0.0343, p-val = 0.8531 |
| **IL-13** | *g*=-0.18; 95%CI: -0.99-0.62; *p*=0.66 | concordant  ↔ in patients | *g*=0.51; 95%CI: -0.02-1.00; *p*=0.04 | **↑ in patients** | 4 | QM(df = 1) = 0.5257, p-val = 0.4684 |
| **IL-18** | *g*=2.61; 95%CI: 1.04-2.19; *p*<0.01 | concordant  ↑ in patients | *g*=2.01; 95%CI: 1.10-2.92; *p*<0.01 | concordant  ↑ in patients | *N/A* | *N/A* |
| **sIL-1RA** | *g*=0.62; 95%CI: 0.04-1.20; *p=*0.04 | concordant  ↑ in patients | *g*=0.50; 95%CI: 0.11-0.88; *p=*0.01 | concordant  ↑ in patients | *N/A* | *N/A* |
| **sIL-2R** | *g*=0.62; 95%CI: 0.18-1.06; *p*=0.01 | concordant  ↑ in patients | *g*=0.69; 95%CI: 0.38-1.00; *p*<0.01 | concordant  ↑ in patients | *N/A* | *N/A* |
| **sIL-6R** | *N/A* | *N/A* | *g*=2.22; 95%CI: 0.67-3.78; *p*=0.01 | concordant  ↑ in patients | *N/A* | *N/A* |
| **TNFα** | *g*=0.70; 95%CI: 0.39-1.01; *p*<0.01 | concordant  ↑ in patients | *g*=0.56; 95%CI: 0.34-0.77; *p*<0.01 | concordant  ↑ in patients | 16 | QM(df = 1) = 0.7546, p-val = 0.3850 |
| **CRP** | *g*=0.66; 95%CI: 0.36-0.96; *p*<0.01 | concordant  ↑ in patients | *g*=0.67; 95%CI: 0.49-0.85; *p*<0.01 | concordant  ↑ in patients | 4 | QM(df = 1) = 0.5723, p-val = 0.4493 |
| **IFNγ** | *g*=0.46; 95%CI: -0.09-1.01; *p*=0.10 | concordant  ↔ in patients | *g*=0.05; 95%CI: -0.33-0.44; *p*=0.78 | concordant  ↔ in patients | 6 | **QM(df = 1) = 8.8421, p-val = 0.0029** |
| **TGFβ** | *g*=0.09; 95%CI: -0.74-0.92; *p*=0.82 | concordant  ↔ in patients | *g*=-0.01; 95%CI: -0.64-0.61; *p*=0.97 | concordant  ↔ in patients | 3 | QM(df = 1) = 0.2712, p-val = 0.6025 |

## Supplementary Table 4: Sensitivity Analyses of lifestyle clinical predictors for mean differences in immune parameters in depression compared with healthy controls.

Results in bold are significant/significantly different from the main analysis.

| **cytokine** | **AGE** | | **BMI** | | **SMOKING** |
| --- | --- | --- | --- | --- | --- |
|  | **meta-regression result and number of studies** | **meta-analysis excluding studies not matched for age and concordance with main analysis** | **meta-regression result and number of studies** | **meta-analysis excluding studies not matched for BMI and concordance with main analysis** | **meta-analysis excluding studies not matched for smoking status and concordance with main analysis** |
| **IL-1α** | N=4, QM(df = 1) = 0.0089, p-val = 0.9250 | concordant  ↑ in patients | N=3, QM(df = 1) = 0.0029, p-val = 0.9574 | not significant | *N/A* |
| **IL-1β** | N=25, QM(df = 1) = 2.0810, p-val = 0.1491 | concordant  ↑ in patients | N=12, QM(df = 1) = 0.0065, p-val = 0.9356 | concordant  ↑ in patients | not significant |
| **IL-2** | N=11, QM(df = 1) = 0.7027, p-val = 0.4019 | concordant  ↑ in patients | N=4, QM(df = 1) = 0.2031, p-val = 0.6522 | **↓ in patients** | not significant |
| **IL-3** | N=3, QM(df = 1) = 0.1308, p-val = 0.7176 | concordant  ↑ in patients | N=4, QM(df = 1) = 0.2031, p-val = 0.6522 | *N/A* | *N/A* |
| **IL-4** | N=9, QM(df = 1) = 0.1088, p-val = 0.7416 | concordant  ↓ in patients | N=3, QM(df = 1) = 0.5215, p-val = 0.4702 | *N/A* | not significant |
| **IL-5** | N=5, QM(df = 1) = 0.5013, p-val = 0.4789 | concordant  ↔ in patients | N=4, QM(df = 1) = 3.0915, p-val = 0.0787 | concordant  ↔ in patients | concordant  ↔ in patients |
| **IL-6** | N=58, QM(df = 1) = 1.5294, p-val = 0.2162 | concordant  ↑ in patients | N=31, QM(df = 1) = 2.2978, p-val = 0.1296 | concordant  ↑ in patients | concordant  ↑ in patients |
| **IL-7** | N=5, QM(df = 1) = 0.0043, p-val = 0.9478 | concordant  ↑ in patients | N=4, QM(df = 1) = 0.2628, p-val = 0.6082 | not significant | not significant |
| **IL-8** | N=9, QM(df = 1) = 0.4041, p-val = 0.5250 | concordant  ↑ in patients | **N=4, QM(df = 1) = 21.3682, p-val < .0001** | **↓ in patients** | not significant |
| **IL-10** | N=18, QM(df = 1) = 0.0553, p-val = 0.8141 | concordant  ↑ in patients | N=7, QM(df = 1) = 0.7617, p-val = 0.3828 | concordant  ↑ in patients | not significant |
| **IL-12** | N=8, QM(df = 1) = 1.1668, p-val = 0.2801 | concordant  ↑ in patients | N=5, QM(df = 1) = 0.0518, p-val = 0.8200 | concordant  ↑ in patients | concordant  ↑ in patients |
| **IL-13** | N=7, QM(df = 1) = 1.0642, p-val = 0.3023 | concordant  ↔ in patients | N=4, QM(df = 1) = 0.1216, p-val = 0.7273 | concordant  ↔ in patients | concordant  ↔ in patients |
| **IL-18** | N=6, QM(df = 1) = 0.0017, p-val = 0.9675 | concordant  ↑ in patients | N=4, QM(df = 1) = 0.1216, p-val = 0.7273 | *N/A* | *N/A* |
| **sIL-1RA** | N=7, QM(df = 1) = 1.1922, p-val = 0.2749 | concordant  ↑ in patients | N=5, QM(df = 1) = 0.0209, p-val = 0.8851 | concordant  ↑ in patients | *N/A* |
| **sIL-2R** | N=10, QM(df = 1) = 0.0359, p-val = 0.8497 | concordant  ↑ in patients | **N=3, QM(df = 1) = 6.1007, p-val = 0.0135** | concordant  ↑ in patients | *N/A* |
| **sIL-6R** | N=8, QM(df = 1) = 0.2305, p-val = 0.6312 | concordant  ↑ in patients | N=3, QM(df = 1) = 0.1152, p-val = 0.7342 | not significant | not significant |
| **TNFα** | N=45, QM(df = 1) = 2.2030, p-val = 0.1377 | concordant  ↑ in patients | N=24, QM(df = 1) = 1.4524, p-val = 0.2281 | concordant  ↑ in patients | concordant  ↑ in patients |
| **CRP** | N=32, QM(df = 1) = 0.8215, p-val = 0.3648 | concordant  ↑ in patients | N=23, QM(df = 1) = 1.4457, p-val = 0.2292 | concordant  ↑ in patients | concordant  ↑ in patients |
| **IFNγ** | N=15, QM(df = 1) = 1.6159, p-val = 0.2037 | concordant  ↔ in patients | **N=7, QM(df = 1) = 6.4419, p-val = 0.0111** | concordant  ↔ in patients | **↑ in patients** |
| **TGFβ** | N=5, QM(df = 1) = 0.2275, p-val = 0.6334 | concordant  ↔ in patients | N=4, QM(df = 1) = 0.1529, p-val = 0.6957 | **↑ in patients** | *N/A* |

## Supplementary Table 5: I^2^ values for each immune parameter (standardised difference in means analysis)

| **Parameter** | **Study N** | **I^2^ value** |
| --- | --- | --- |
| IL1α | 4 | 86.6 |
| IL1β | 26 | 92.5 |
| IL2 | 12 | 94.0 |
| IL3 | 3 | 47.6 |
| IL4 | 10 | 90.3 |
| IL5 | 5 | 58.5 |
| IL6 | 62 | 92.1 |
| IL7 | 5 | 73.7 |
| IL8 | 9 | 92.4 |
| IL10 | 19 | 90.6 |
| IL12 | 9 | 90.1 |
| IL13 | 7 | 90.4 |
| IL18 | 6 | 92.4 |
| IL1RA | 7 | 71.7 |
| IL2R | 10 | 70.6 |
| IL6R | 8 | 99.0 |
| TNFα | 48 | 91.0 |
| CRP | 35 | 87.1 |
| IFNγ | 16 | 90.1 |
| TGFβ | 5 | 90.6 |

## Supplementary Table 6: Quality assessment of studies included in meta-analysis.

The Newcastle Ottawa Scale considers study quality based on three domains (see Appendix 1 for further details): 1) Quality of subject ‘selection’ (cases and controls, maximum 4 points); 2) Quality of ‘comparability’ of cases and controls (defined a priori as matching for age and BMI, maximum 2 points); 3) Quality of ‘exposure’ (defined as use of a standardised assay for measuring inflammatory markers in the blood, and following a standardised process, maximum 2 points). Item ‘non-response rate’ for Quality of exposure in the scale was not applicable. A maximum of 8 points was therefore considered.

Thresholds for converting NOS rating to Agency for Healthcare Research and Quality standards (good, fair, poor). *Good – 3 or 4 stars in Selection AND 1 or 2 in Comparability AND 1 or 2 in Exposure. Fair – 2 stars in Selection AND 1 or 2 in Comparability AND 1 or 2 in Exposure. Poor – 0 or 1 in stars in selection OR 0 in Comparability OR 0 or 1 in Exposure.*

| **Author/Year** | **Selection** | | | | **Comparability** | | **Exposure** | | **Score**  **/8** | **Quality Rating** |
| --- | --- | --- | --- | --- | --- | --- | --- | --- | --- | --- |
|  | **1** | **2** | **3** | **4** | **Age** | **BMI** | **1** | **2** |  |  |
| Alcocer-Gomez et al., 2014 | 1 | 0 | 0 | 1 | 1 | 1 | 1 | 1 | 6 | Fair |
| Alesci et al., 2005 | 1 | 0 | 1 | 1 | 1 | 1 | 1 | 1 | 7 | Good |
| Ali et al., 2017 | 1 | 1 | 1 | 1 | 1 | 1 | 1 | 0 | 7 | Good |
| Bai et al., 2014 | 1 | 0 | 0 | 1 | 1 | 1 | 1 | 0 | 5 | Fair |
| Basterzi et al., 2005 | 1 | 0 | 0 | 1 | 1 | 0 | 1 | 1 | 5 | Fair |
| Berk et al., 1997 | 1 | 0 | 1 | 0 | 1 | 1 | 1 | 0 | 5 | Fair |
| Boettger et al., 2010 | 1 | 0 | 1 | 1 | 1 | 1 | 1 | 0 | 6 | Good |
| Brambilla et al., 1998 | 1 | 0 | 1 | 1 | 1 | 0 | 1 | 1 | 6 | Good |
| Camardese, 2011 | 1 | 0 | 0 | 1 | 1 | 0 | 1 | 1 | 5 | Fair |
| Cassano et al., 2017 | 1 | 0 | 1 | 1 | 1 | 1 | 1 | 0 | 6 | Good |
| Chamberlain et al., 2018 | 1 | 0 | 1 | 1 | 1 | 1 | 1 | 1 | 7 | Good |
| Charlton et al., 2018 | 1 | 0 | 1 | 0 | 1 | 1 | 1 | 1 | 6 | Fair |
| Chavda et al., 2011 | 1 | 0 | 1 | 1 | 1 | 0 | 0 | 0 | 4 | Poor |
| Chen et al., 2017 | 1 | 0 | 0 | 1 | 1 | 0 | 1 | 1 | 5 | Fair |
| Crnkovic et al., 2012 | 1 | 0 | 1 | 0 | 0 | 1 | 1 | 1 | 5 | Fair |
| Dannehl et al., 2014 | 1 | 0 | 0 | 1 | 1 | 1 | 1 | 1 | 6 | Fair |
| Davami et al., 2016 | 1 | 0 | 0 | 1 | 1 | 1 | 1 | 0 | 5 | Fair |
| Dhabhar et al., 2009 | 1 | 0 | 1 | 1 | 1 | 0 | 1 | 1 | 6 | Good |
| Dinan et al., 2009 | 1 | 0 | 0 | 0 | 0 | 0 | 1 | 1 | 3 | Poor |
| Diniz et al., 2010 | 1 | 0 | 0 | 1 | 1 | 0 | 1 | 0 | 4 | Fair |
| Dome et al., 2009 | 1 | 0 | 0 | 0 | 1 | 1 | 1 | 0 | 4 | Poor |
| Dunjic-Kostic et al., 2013 | 1 | 0 | 0 | 1 | 1 | 1 | 1 | 1 | 6 | Fair |
| Elderkin-Thompson et al., 2012 | 1 | 0 | 1 | 0 | 1 | 1 | 1 | 1 | 6 | Fair |
| Eller et al., 2008 | 1 | 0 | 0 | 1 | 1 | 0 | 1 | 1 | 5 | Fair |
| Elomaa et al., 2012 | 1 | 1 | 1 | 1 | 1 | 1 | 1 | 0 | 7 | Good |
| Euteneuer et al., 2012 | 1 | 0 | 0 | 1 | 1 | 1 | 1 | 0 | 5 | Fair |
| Euteneuer et al., 2011 | 1 | 0 | 0 | 1 | 1 | 1 | 1 | 0 | 5 | Fair |
| Fan et al., 2017 | 1 | 0 | 1 | 0 | 1 | 0 | 1 | 1 | 6 | Fair |
| Fornaro et al., 2011 | 1 | 0 | 0 | 1 | 1 | 0 | 1 | 1 | 5 | Fair |
| Fornaro et al., 2013 | 1 | 0 | 0 | 1 | 1 | 0 | 1 | 1 | 5 | Fair |
| Frodl et al., 2012 | 1 | 0 | 1 | 1 | 1 | 1 | 1 | 0 | 6 | Good |
| Frommberger, 1997 | 1 | 0 | 0 | 1 | 1 | 0 | 1 | 1 | 5 | Fair |
| Gazal et al., 2015 | 1 | 1 | 1 | 0 | 1 | 0 | 1 | 1 | 6 | Good |
| Goyal et al., 2018 | 1 | 0 | 0 | 1 | 1 | 1 | 1 | 1 | 6 | Fair |
| Grosse et al., 2016 | 1 | 0 | 1 | 1 | 1 | 1 | 1 | 0 | 6 | Good |
| Hafner et al., 2008 | 1 | 0 | 0 | 1 | 1 | 1 | 1 | 0 | 5 | Fair |
| Hernandez et al., 2008 | 1 | 0 | 1 | 1 | 1 | 1 | 1 | 1 | 7 | Good |
| Ho et al., 2017 | 1 | 0 | 1 | 1 | 1 | 1 | 1 | 1 | 7 | Good |
| Hocaoglu et al., 2012 | 1 | 0 | 0 | 1 | 1 | 0 | 1 | 1 | 5 | Fair |
| Hosseini et al., 2007 | 1 | 0 | 1 | 0 | 1 | 0 | 1 | 1 | 5 | Fair |
| Huang et al., 2007 | 1 | 0 | 0 | 1 | 1 | 1 | 1 | 1 | 6 | Fair |
| Hughes et al., 2012 | 1 | 0 | 1 | 1 | 1 | 1 | 1 | 0 | 6 | Good |
| Hung, 2007 | 1 | 0 | 0 | 0 | 1 | 1 | 1 | 1 | 5 | Poor |
| Jozuga et al., 2003 | 1 | 0 | 0 | 0 | 1 | 0 | 1 | 1 | 4 | Poor |
| Kagaya et al., 2001 | 1 | 0 | 0 | 1 | 1 | 0 | 1 | 1 | 5 | Fair |
| Kageyama et al., 2017 | 1 | 0 | 1 | 1 | 1 | 0 | 1 | 0 | 5 | Good |
| Kahl et al., 2015 | 1 | 0 | 0 | 1 | 1 | 1 | 1 | 1 | 6 | Fair |
| Karlovic et al., 2012 | 1 | 0 | 0 | 1 | 1 | 0 | 1 | 1 | 5 | Fair |
| Keri et al., 2014 | 1 | 0 | 1 | 1 | 1 | 1 | 1 | 1 | 7 | Good |
| Kim et al., 2002 | 1 | 0 | 1 | 1 | 1 | 1 | 1 | 1 | 7 | Good |
| Kling et al., 2007 | 1 | 0 | 1 | 1 | 1 | 1 | 1 | 0 | 6 | Good |
| Kokai et al., 2002 | 1 | 0 | 0 | 1 | 1 | 0 | 1 | 1 | 5 | Fair |
| Kubera et al., 2000 | 1 | 0 | 0 | 1 | 1 | 0 | 1 | 1 | 5 | Fair |
| Lanquillon et al., 2000 | 1 | 1 | 0 | 0 | 1 | 0 | 1 | 1 | 5 | Fair |
| Lee et al., 2006 | 1 | 0 | 1 | 1 | 1 | 1 | 1 | 1 | 7 | Good |
| Lee et al., 2009 | 1 | 0 | 0 | 0 | 1 | 1 | 1 | 0 | 4 | Poor |
| Lehto et al., 2010 | 1 | 1 | 1 | 1 | 1 | 1 | 1 | 1 | 8 | Good |
| Leo et al., 2006 | 1 | 0 | 0 | 1 | 1 | 1 | 1 | 1 | 6 | Fair |
| Maes et al., 1997 | 1 | 0 | 0 | 1 | 1 | 0 | 1 | 1 | 5 | Fair |
| Maes et al., 1990 | 1 | 0 | 0 | 1 | 1 | 0 | 1 | 1 | 5 | Fair |
| Manoharan et al., 2016 | 1 | 0 | 1 | 1 | 1 | 1 | 1 | 1 | 7 | Good |
| Marques-Deak et al., 2007 | 1 | 0 | 0 | 1 | 1 | 1 | 1 | 1 | 6 | Fair |
| Merendino et al., 2002 | 1 | 0 | 0 | 0 | 1 | 0 | 1 | 0 | 3 | Poor |
| Mikova et al., 2001 | 1 | 1 | 0 | 0 | 1 | 0 | 1 | 1 | 5 | Fair |
| Miller et al., 2005 | 1 | 0 | 1 | 1 | 1 | 1 | 1 | 1 | 7 | Good |
| Mota et al., 2013 | 1 | 0 | 1 | 1 | 1 | 1 | 1 | 1 | 7 | Good |
| Motivala et al., 2005 | 1 | 0 | 1 | 1 | 1 | 1 | 1 | 1 | 7 | Good |
| Munjiza et al., 2018 | 1 | 1 | 0 | 1 | 1 | 0 | 1 | 1 | 6 | Good |
| Narita et al., 2006 | 1 | 0 | 0 | 1 | 1 | 1 | 1 | 1 | 6 | Fair |
| Nunes et al., 2012 | 1 | 0 | 0 | 0 | 1 | 1 | 1 | 0 | 4 | Poor |
| O’Brien et al., 2007 | 1 | 0 | 0 | 1 | 1 | 0 | 1 | 1 | 5 | Fair |
| O’Brien et al., 2006 | 1 | 0 | 0 | 1 | 1 | 0 | 1 | 1 | 5 | Fair |
| O’Donovan et al., 2013 | 1 | 0 | 1 | 1 | 1 | 0 | 1 | 1 | 6 | Good |
| Oglodek et al., 2018 | 1 | 0 | 1 | 0 | 1 | 0 | 1 | 1 | 5 | Fair |
| Owen et al., 2008 | 1 | 0 | 0 | 0 | 1 | 0 | 1 | 0 | 3 | Poor |
| Pavon et al., 2006 | 1 | 0 | 1 | 1 | 1 | 0 | 1 | 1 | 6 | Good |
| Pike et al., 2006 | 1 | 0 | 1 | 1 | 1 | 1 | 1 | 1 | 7 | Good |
| Piletz et al., 2009 | 1 | 0 | 1 | 1 | 1 | 1 | 1 | 1 | 7 | Good |
| Rapaport et al., 1996 | 1 | 0 | 0 | 0 | 1 | 0 | 1 | 0 | 3 | Poor |
| Rawdin et al., 2013 | 1 | 0 | 1 | 1 | 1 | 1 | 1 | 1 | 7 | Good |
| Rief et al., 2001 | 1 | 0 | 0 | 1 | 1 | 1 | 1 | 1 | 6 | Fair |
| Rivazi et al., 2016 | 1 | 0 | 1 | 1 | 1 | 0 | 1 | 1 | 6 | Good |
| Rudolf et al., 2014 | 1 | 0 | 1 | 1 | 1 | 1 | 1 | 1 | 7 | Good |
| Rudzki et al., 2017 | 1 | 0 | 0 | 0 | 1 | 1 | 1 | 1 | 5 | Poor |
| Rybka et al., 2013 | 1 | 0 | 1 | 1 | 1 | 1 | 1 | 1 | 7 | Good |
| Savitz et al., 2015 | 1 | 0 | 0 | 0 | 1 | 1 | 1 | 1 | 5 | Poor |
| Schlatter et al., 2004 | 1 | 0 | 0 | 1 | 1 | 0 | 1 | 1 | 5 | Fair |
| Schmidt et al., 2014 | 1 | 0 | 1 | 1 | 1 | 0 | 1 | 0 | 5 | Good |
| Seidel et al., 1995 | 1 | 0 | 0 | 1 | 1 | 0 | 1 | 1 | 5 | Fair |
| Shen et al., 2010 | 1 | 0 | 0 | 1 | 1 | 0 | 1 | 1 | 5 | Fair |
| Simon et al., 2008 | 1 | 0 | 1 | 1 | 1 | 0 | 0 | 0 | 4 | Poor |
| Sluzewska et al., 1996 | 1 | 0 | 1 | 1 | 1 | 0 | 1 | 1 | 6 | Good |
| Sowa-Kucma et al., 2018 | 1 | 0 | 0 | 1 | 1 | 1 | 1 | 1 | 6 | Fair |
| Sugimoto et al., 2018 | 1 | 0 | 1 | 1 | 1 | 0 | 1 | 0 | 5 | Good |
| Sutcigil et al., 2007 | 1 | 0 | 0 | 1 | 1 | 0 | 1 | 1 | 5 | Fair |
| Thomas et al., 2005 | 1 | 0 | 0 | 1 | 1 | 0 | 1 | 0 | 4 | Fair |
| Tuglu et al., 2003 | 1 | 1 | 0 | 1 | 1 | 0 | 1 | 1 | 6 | Good |
| Vaccarino et al., 2008 | 1 | 0 | 1 | 0 | 1 | 1 | 1 | 0 | 5 | Fair |
| Wang et al., 2018 | 1 | 0 | 0 | 0 | 1 | 1 | 1 | 1 | 5 | Poor |
| Wiener et al., 2017 | 1 | 0 | 0 | 0 | 1 | 0 | 1 | 0 | 3 | Poor |
| Xia et al., 2018 | 1 | 0 | 0 | 0 | 1 | 1 | 1 | 0 | 4 | Poor |
| Yang et al., 2007 | 1 | 0 | 0 | 0 | 1 | 1 | 1 | 1 | 5 | Poor |
| Yoshimura et al., 2009 | 1 | 0 | 0 | 0 | 1 | 0 | 1 | 1 | 4 | Poor |
| Yoshimura et al., 2010 | 1 | 0 | 0 | 0 | 1 | 0 | 1 | 1 | 4 | Poor |
| Zincir et al., 2016 | 1 | 0 | 0 | 1 | 1 | 0 | 1 | 1 | 5 | Fair |
| Zoga et al., 2014 | 1 | 0 | 0 | 1 | 1 | 1 | 1 | 1 | 6 | Fair |
| Zou et al., 2018 | 1 | 0 | 0 | 1 | 1 | 1 | 1 | 1 | 6 | Fair |

## Supplementary Table 7: Full Summary of Findings

Results for sensitivity analyses are marked with by a **^+^** sign if all results if one category (e.g. Psychiatric clinical predictors) are concordant with the main analysis for that immune parameter, or by an * if one or more of the results within that category are non-significant or discordant from the main analysis.

|  | **Meta-analysis of mean differences in immune parameters in depression compared with healthy controls** | | | | | | | **Meta-analysis of variability** |
| --- | --- | --- | --- | --- | --- | --- | --- | --- |
| **cytokine** | **Main mean meta-analysis** | **psychiatric clinical predictors** | **lifestyle clinical predictors** | **influence of sample type (plasma/serum and fresh/frozen)** | **influence of residual skew** | **Sensitivity analysis excluding poor quality studies** | **Exclusion of outlier for publication bias** | **CVR** |
| **IL-1α** | ↑ in patients | ↑ in patients^+^ | ↑ in patients * | N/A | N/A | N/A | ↑ in patients^+^ | ↔ |
| **IL-1β** | ↑ in patients | ↑ in patients^+^ | ↑ in patients * | ↑ in patients | ↔ | ↑ in patients^+^ | ↑ in patients^+^ | ↔ |
| **IL-2** | ↑ in patients | ↑ in patients^+^ | discordant | ↑ in patients | ↑ in patients^+^ | ↔ | ↑ in patients^+^ | ↑ variability in patients |
| **IL-3** | ↑ in patients | ↑ in patients^+^ | ↑ in patients^+^ | N/A | N/A | N/A | ↑ in patients^+^ | ↔ |
| **IL-4** | ↓ in patients | ↓ in patients^+^ | ↓ in patients* | ↓ in patients | ↓ in patients^+^ | ↓ in patients^+^ | ↓ in patients^+^ | ↔ |
| **IL-5** | ↔ | ↑ in patients^*^ | ↔ in patients^+^ | ↔ | N/A | ↔ in patients^+^ | ↔ in patients^+^ | ↔ |
| **IL-6** | ↑ in patients | ↑ in patients^+^ | ↑ in patients^+^ | ↑ in patients | ↑ in patients^+^ | ↑ in patients^+^ | ↑ in patients^+^ | ↔ |
| **IL-7** | ↑ in patients | ↑ in patients^+^ | ↑ in patients^*^ | ↔ | N/A | ↔ | ↑ in patients^+^ | ↔ |
| **IL-8** | ↑ in patients | ↑ in patients^+^ | discordant | ↔ | discordant ↓ in patients | ↔ | ↑ in patients^+^ | ↔ |
| **IL-10** | ↑ in patients | ↑ in patients^*^ | ↑ in patients^*^ | ↔ | ↑ in patients^+^ | ↑ in patients^+^ | ↑ in patients^+^ | ↔ |
| **IL-12** | ↑ in patients | ↑ in patients^+^ | ↑ in patients^+^ | ↑ in patients | ↑ in patients^+^ | ↑ in patients^+^ | ↑ in patients^+^ | ↓ variability in patients |
| **IL-13** | ↔ | ↑ in patients when significant | ↔ in patients^+^ | ↔ | N/A | ↑ in patients when significant | ↔ in patients^+^ | ↓ variability in patients |
| **IL-18** | ↑ in patients | ↑ in patients^+^ | ↑ in patients^+^ | ↑ in patients | ↑ in patients^+^ | ↑ in patients^+^ | ↑ in patients^+^ | ↔ |
| **sIL-1RA** | ↑ in patients | ↑ in patients^+^ | ↑ in patients^+^ | ↑ in patients | ↑ in patients^+^ | ↔ | ↑ in patients^+^ | ↔ |
| **sIL-2R** | ↑ in patients | ↑ in patients^+^ | ↑ in patients^+^ | ↑ in patients | ↑ in patients^+^ | ↑ in patients^+^ | ↑ in patients^+^ | ↓ variability in patients |
| **sIL-6R** | ↑ in patients | ↑ in patients^+^ | ↑ in patients^+^ | ↔ | ↑ in patients^+^ | ↔ | ↔ | ↔ |
| **TNFα** | ↑ in patients | ↑ in patients^+^ | ↑ in patients^+^ | ↑ in patients | ↑ in patients^+^ | ↑ in patients^+^ | ↑ in patients^+^ | ↔ |
| **CRP** | ↑ in patients | ↑ in patients^+^ | ↑ in patients^+^ | ↑ in patients | ↑ in patients^+^ | ↑ in patients^+^ | ↑ in patients^+^ | ↓ variability in patients |
| **IFNγ** | ↔ | ↔ in patients^+^ | ↑ in patients when significant | ↔ | ↔ in patients^+^ | ↔ in patients^+^ | ↔ in patients^+^ | ↓ variability in patients |
| **TGFβ** | ↔ | ↔ in patients^+^ | ↑ in patients when significant | ↔ | ↑ in patients | ↔ in patients^+^ | ↔ in patients^+^ | ↑ variability in patients |

# SUPPLEMENTARY APPENDIX 1: Newcastle Ottawa Quality assessment scale for case control studies

**SELECTION**

**1) Is the case definition adequate?**

*a) yes, with independent validation (1 point)*

b) yes, e.g. record linkage or based on self-reports

c) no description

**2) Representativeness of the cases**

*a) consecutive or obviously representative series of cases (1 point)*

b) potential for selection biases or not stated

**3) Selection of Controls**

*a) community controls (1 point)*

b) hospital controls

c) no description

**4) Definition of Controls**

*a) no history of psychiatric or medical disease (1 point)*

b) any history of psychiatric or medical comorbidity

**COMPARABILITY**

**1) Comparability of cases and controls based on the design or analysis**

*a) study controls for age (1 point)*

*b) study controls for BMI (1 point)*

**EXPOSURE: immune factor measurement**

**1) Assay**

*a) specifies assay type, brand/model (1 point)*

*b) standardised protocol for sample collection (1 point)*

e) no description or no standardised protocol

**2) Same method of ascertainment for cases and controls**

*a) yes (1 point)*

b) no

# Supplementary references

1. *EndNote* [computer program]. Version X8: Clarivate Analytics; 2018.

2. *WebPlotDigitizer* [computer program]. Version 3.102016.

3. Viechtbauer W. Conducting meta-analyses in R with the metafor package. *Journal of Statistical Software.* 2010;36(3):1-48.

4. *R: A language and environment for statistical computing [Software]* [computer program]. Vienna, Austria: R Foundation for Statistical Computing; 2017.

5. Altman DG, Bland JM. Detecting skewness from summary information. *British Medical Journal.* 1996;313(7066):1200-1201.

6. Higgins JPT, Green S, Cochrane Collaboration. *Cochrane handbook for systematic reviews of interventions.* Chichester, England ; Hoboken, NJ: Wiley-Blackwell; 2008.

7. Brugger SP, Howes OD. Heterogeneity and homogeneity of regional brain structure in schizophrenia: a meta-analysis. *JAMA psychiatry.* 2017;74(11):1104-1111.

8. Pillinger T, Osimo EF, Brugger S, Mondelli V, McCutcheon RA, Howes OD. A Meta-analysis of Immune Parameters, Variability, and Assessment of Modal Distribution in Psychosis and Test of the Immune Subgroup Hypothesis. *Schizophrenia Bulletin.* 2018:sby160.

9. Stang A. Critical evaluation of the Newcastle-Ottawa scale for the assessment of the quality of nonrandomized studies in meta-analyses. *European journal of epidemiology.* 2010;25(9):603-605.

10. Camardese G, Pizi G, Marino M, et al. Alterazioni delle risposte immuno-infiammatorie nei pazienti affetti da disturbo depressivo maggiore. *Giorn Ital Psicopat.* 2011;17:396-403.
